# Identification and Characterization of Carboxylesterases from *Brachypodium distachyon* Deacetylating Trichothecene Mycotoxins

**DOI:** 10.3390/toxins8010006

**Published:** 2015-12-25

**Authors:** Clemens Schmeitzl, Elisabeth Varga, Benedikt Warth, Karl G. Kugler, Alexandra Malachová, Herbert Michlmayr, Gerlinde Wiesenberger, Klaus F. X. Mayer, Hans-Werner Mewes, Rudolf Krska, Rainer Schuhmacher, Franz Berthiller, Gerhard Adam

**Affiliations:** 1 Department of Applied Genetics and Cell Biology, University of Natural Resources and Life Sciences, Vienna (BOKU), Konrad-Lorenz-Strasse 24, 3430 Tulln, Austria; herbert.michlmayr@boku.ac.at (H.M.); gerlinde.wiesenberger@boku.ac.at (G.W.); gerhard.adam@boku.ac.at (G.A.); 2 Center for Analytical Chemistry, Department of Agrobiotechnology (IFA-Tulln), University of Natural Resources and Life Sciences, Vienna (BOKU), Konrad-Lorenz-Strasse 20, 3430 Tulln, Austria; elisabeth.varga@boku.ac.at (E.V.); alexandra.malachova@boku.ac.at (A.M.); rudolf.krska@boku.ac.at (R.K.); rainer.schuhmacher@boku.ac.at (R.S.); franz.berthiller@boku.ac.at (F.B.); 3 Christian Doppler Laboratory for Mycotoxin Metabolism, Konrad-Lorenz-Strasse 20, 3430 Tulln, Austria; 4 Plant Genome and Systems Biology, Helmholtz Zentrum München, Ingolstädter Landstrasse 1, 85764 Neuherberg, Germany; karl.kugler@helmholtz-muenchen.de (K.G.K.); kmayer@helmholtz-muenchen.de (K.F.X.M.); 5 Genome oriented Bioinformatics, Technische Universität München, Wissenschaftszentrum Weihenstephan, Am Forum 1, 85354 Freising, Germany; w.mewes@wzw.tum.de

**Keywords:** *Fusarium graminearum*, trichothecene metabolism, 3-acetyl-deoxynivalenol, 15-acetyl-deoxynivalenol, monocot, enzymatic cleavage

## Abstract

Increasing frequencies of 3-acetyl-deoxynivalenol (3-ADON)-producing strains of *Fusarium graminearum* (3-ADON chemotype) have been reported in North America and Asia. 3-ADON is nearly nontoxic at the level of the ribosomal target and has to be deacetylated to cause inhibition of protein biosynthesis. Plant cells can efficiently remove the acetyl groups of 3-ADON, but the underlying genes are yet unknown. We therefore performed a study of the family of candidate carboxylesterases (CXE) genes of the monocot model plant *Brachypodium distachyon*. We report the identification and characterization of the first plant enzymes responsible for deacetylation of trichothecene toxins. The product of the *BdCXE29* gene efficiently deacetylates T-2 toxin to HT-2 toxin, NX-2 to NX-3, both 3-ADON and 15-acetyl-deoxynivalenol (15-ADON) into deoxynivalenol and, to a lesser degree, also fusarenon X into nivalenol. The BdCXE52 esterase showed lower activity than BdCXE29 when expressed in yeast and accepts 3-ADON, NX-2, 15-ADON and, to a limited extent, fusarenon X as substrates. Expression of these *Brachypodium* genes in yeast increases the toxicity of 3-ADON, suggesting that highly similar genes existing in crop plants may act as susceptibility factors in Fusarium head blight disease.

## 1. Introduction

*Brachypodium distachyon* is a monocot grass species closely related to economically-important crops, like bread wheat (*Triticum aestivum*), barley (*Hordeum vulgare*) and rice (*Oryza sativa*). Its rapid lifecycle, simple growth requirements, small and fully-sequenced genome and the availability of efficient transformation protocols make *B. distachyon* an important model plant for more complex cereals. As an emerging pathosystem, it is also used for studying the interaction with *Fusarium graminearum* [1,2,3,4], which causes the disease Fusarium head blight (FHB) in cereals. During infection of the host plant, *Fusarium* species produce trichothecene mycotoxins, like deoxynivalenol (DON), potentially being a risk for humans and livestock if entering the food chain, constituting a major agronomic problem [5]. The most important Fusarium head blight-causing members of the *F. graminearum* species complex [6], *F. graminearum* and *Fusarium asiaticum*, can be grouped into so-called chemotypes depending on the trichothecene toxin they mainly produce in culture. Strains produce either nivalenol (NIV) or DON; the DON producers can be sub-grouped into 3-acetyl-DON (3-ADON) and 15-acetyl-DON (15-ADON) chemotypes. It has been shown that the allele of the carboxylesterase encoded by *TRI8* determines which acetyl group is cleaved off from the common precursor 3,15-di-acetyl-DON, resulting in the production of 3-ADON or 15-ADON [7]. The NIV chemotype is determined by the allele of the *TRI13* encoding a cytochrome P450 [8]. DON chemotype strains contain a loss of function allele; the C4 is therefore not hydroxylated. *F. graminearum* strains typically produce type B trichothecenes possessing a keto group at C8. Recently, a new chemotype was described (caused by an allele of *TRI1*) producing the NX-toxins, which lack a C8-keto group [9]. Interestingly, all currently-known isolates synthesizing this new toxin are of the “3-ADON” chemotype. *In planta*, the NX-2 toxin is deacetylated to NX-3 [9]. Trichothecenes are known as potent protein synthesis inhibitors, but it has been shown that 3-ADON is about two orders of magnitude less toxic at the ribosomal level than DON [10]. Nevertheless, in recent years, at least two independent chemotype shifts occurred in North America and Asia leading to the increased prevalence of 3-ADON strains [11,12,13]. The reason for this development is unclear. We have recently shown that wheat cells are capable of efficiently deacetylating 3-ADON, 15-ADON and 3,15-diADON [14]. The genes responsible for this activity are unknown.

Carboxylesterases (CXE, E.C.3.1.1.1) belong to the α/β-hydrolase superfamily usually consisting of an eight β-strand core connected by loops and α-helices [15]. A hallmark of all CXEs is the active site, which consists of the catalytic triad, including an active site serine embedded in a GXSXG motif, an acidic residue (mostly aspartate in plants and glutamate in mammals) and a histidine. Additionally, an important feature is the highly-conserved HGG box, forming an oxyanion hole involved in the stabilization of the substrate-enzyme intermediate during hydrolysis [15]. The best-studied carboxylesterases are probably the human CES1 and CES2 proteins performing various functions in xenobiotic, drug and lipid metabolism [16]. Evidence for plant carboxylesterases playing a role in deacetylating ADONs was lacking, and studies on members of this large gene family in plants are generally scarce. Only the CXE gene family of *Arabidopsis thaliana* consisting of 20 members, has been described [17]. Four gene products have been biochemically characterized as active carboxylesterases. AtCXE8 and AtCXE9 show carboxylesterase activity with the standard esterase substrates 4-nitrophenyl acetate and 4-nitrophenyl butyrate [18]. AtCXE12 hydrolyzes the pro-herbicide methyl-2,4-dichlorophenoxyacetate into the active compound 2,4-dichlorophenoxyacetic acid [19]. AtCXE18 accepts esters based on methylumbelliferone, similar to pig liver esterase [20]. Interestingly, three more family members were identified as gibberellin receptors and have been (re-)classified as hormone-sensitive lipases (HSL; EC 3.1.1.79) [21]. Although being closely related to CXEs, all three *A. thaliana* gibberellin receptor genes (*GID*) and the *GID1* from rice can be distinguished from CXEs by not possessing an active site histidine, but a conserved arginine essential for maintaining the gibberellin binding activity [21]. However, for most *Arabidopsis* CXE genes, the function is still unknown. In other plant orders, CXEs are important for the activation of plant signaling compounds, including salicylic acid and jasmonic acid from their respective methyl esters, and regulating activity and transport during natural product synthesis [22].

Recently, we showed that both 15-ADON and 3-ADON are rapidly deacetylated by wheat cells [14]. DON, a virulence factor of *Fusarium*, can be metabolized into DON-3-*O*-glucoside (DON-3G), which is the major detoxification pathway of DON in *Arabidopsis* [23] and wheat [24]. Increased Fusarium resistance of wheat overexpressing a barley glucosyltransferase has been demonstrated [25]. We recently reported direct glycosylation of 15-ADON to 15-ADON-3-*O*-glucoside (15-ADON3G) [14]. 3-ADON is nearly unable to inhibit ribosomes, but if deacetylation of 3-ADON occurs more rapidly than glycosylation, toxic DON may accumulate intracellularly.

The objective of this study was to identify genes in the model plant *B. distachyon* that encode enzymes deacetylating trichothecene mycotoxins. Our working hypothesis is that members of the carboxylesterase gene family might be responsible for the enzymatic cleavage. The most significant outcome of our experiments is the identification of plant genes that increase the toxicity of 3-ADON and that might play a role as susceptibility factors in crops.

## 2. Results and Discussion

### 2.1. Brachypodium distachyon Deacetylates 3-ADON and 15-ADON

**Figure 1:**
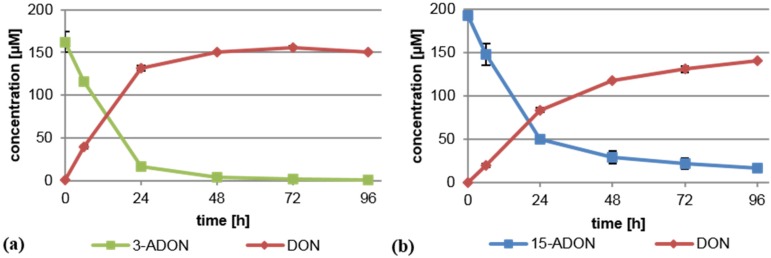
Deacetylation of (**a**) 3-acetyl-deoxynivalenol (3-ADON) and (**b**) 15-acetyl-deoxynivalenol (15-ADON) to deoxynivalenol (DON) by *B. distachyon* (Bd21) cell suspension cultures. Shown is the concentration measured in the medium.

In order to test whether *B. distachyon* exhibits a similar deacetylating capacity as wheat, we performed experiments with a Bd21 suspension culture under identical experimental conditions as previously described [14]. In brief, a dense *B. distachyon* cell suspension culture was supplemented separately with 75 mg/L (221 µM) 3-ADON or 15-ADON, and its metabolism was monitored by analyzing the supernatant via LC-MS/MS. As shown previously for wheat, 3-ADON and 15-ADON were both rapidly converted into DON by the *Brachypodium* cells (Figure 1). No 3-ADON was detectable after 96 h in the medium. Metabolism of 15-ADON was slower and less complete, but still, most of the 15-ADON was deacetylated to DON. The high DON values in the supernatant could indicate that either extracellular enzymes deacetylating 3-ADON and 15-ADON exist or that they are intracellularly deacetylated and DON is efficiently transported out of the cells. We conclude that Bd21 is a suitable model for the analysis of the deacetylation of trichothecenes.

### 2.2. The CXE Gene Family of B. Distachyon

Based on the working hypothesis that certain carboxylesterase gene products might deacetylate trichothecenes, we started to characterize the gene family of *B. distachyon* with the goal to identify a protein acting on 3-ADON. The previously-reported CXE gene family of *Arabidopsis* and two putative rice CXEs (OsCXE1, BAB44070.1; OsCXE5, BAB90534.1) were used for a protein BLAST search against *B. distachyon* using the “MIPS (Munich Information Center for Protein Sequences) PlantsDB v1.2” (http://pgsb.helmholtz-muenchen.de/plant/brachypodium/index.jsp) [26], yielding 51 putative carboxylesterase genes (*BdCXE*). Recent reassessment utilizing Phytozome (phytozome.jgi.doe.gov) [27], including an updated annotation (v2.1), increased the total amount of putative carboxylesterases to 56 (Table 1). The predicted loci of the putative *BdCXE*s are dispersed throughout all five chromosomes of *B. distachyon*. Chromosome 1 harbors most of the predicted CXEs (25), chromosome 5 the least (two). The predicted CXEs are often found in clusters of up to seven genes. Interestingly, most of these gene clusters cannot be explained by simple tandem duplication of ancestral genes. As indicated in Table 1, some genes face in different directions than the rest of the cluster. For example, Bradi3g38080.1 and Bradi38090.2 are highly similar genes with 82% identity found next to each other on the genome, but show a tail-to-tail orientation. Some gene models were modified between annotations v1.2 and v2.1 while the experimental work was ongoing; therefore, both annotations are included in Table 1. The length of the predicted open reading frames range from 924 to 2643 bp, with an average of about 1100 bp, which is similar to the average length of the previously-published *AtCXE* genes (1041 bp). Most of the CXE gene models do not contain any intron; six contain one intron, and four gene models contain two or more introns (Table 1).

Bradi1g06240.1 and Bradi1g19715.1 were predicted to be unusually large putative CXEs, being 1557 and 1935 bp, respectively. Bradi1g06240.1 has a more than 600-bp *C*-terminal insertion potentially encoding a Myb/SANT-like deoxyribonucleic acid (DNA)-binding domain [28] not conserved in other cereal homologs. Crosschecking utilizing annotation v2.1 revealed that the annotations of Bradi1g06240.1 and Bradi1g19715.1 were very likely prediction artifacts. Bradi1g06240.1 has been updated to Bradi1g06241.1 without the DNA-binding domain, and Bradi1g19715.1 was spilt into two separate genes both encoding putative carboxylesterases (Table 1). In contrast, the annotation of locus Bradi1g45925.1 has been altered to include now a NADH dehydrogenase-like *N*-terminal domain encompassing 11 introns (new Bradi1g45921.1). However, this new gene model is most likely also an artificial fusion of neighboring genes. Furthermore, the annotations of seven more gene models (Bradi1g19750.1, Bradi1g56817.1, Bradi3g38090.1, Bradi4g12370.1, Bradi4g32080.1, Bradi4g32330.1, Bradi4g39410.1) have been altered or amended by a second splice variant, and five new gene models have been introduced (Bradi1g38325.1, Bradi1g48173.1, Bradi1g48203.1, Bradi1g50705.1, Bradi4g24773.1). Bradi1g56817.2 contains a “1-nt intron” to maintain higher similarity to other genes after the insertion of one nucleotide. Theoretically, the predicted protein could exist in low amounts in the case of a+1 translational frameshift [29], but most likely, it is an inactive gene due to the frameshift in the *C*-terminus.

In total, eleven gene models do not contain a complete active site. Bradi4g39410.1 (but not Bradi4g39410.2) does not have the oxyanion hole. Bradi1g67930.1, Bradi3g21747.1 and Bradi1g48173.1 do not possess the complete GXSXG motif. In Bradi1g74240.1 and Bradi4g32330, the acidic residue of the active site is replaced by a cysteine. Bradi1g19716.1, Bradi1g56817.1 (but not Bradi1g56817.2), Bradi1g67930.1, Bradi2g25600.1 and Bradi3g42207.1 are missing the active site histidine in the putative active site. Those predicted CXEs therefore are unlikely to encode active carboxylesterases. Furthermore, Bradi2g25600.1 seems to be a gibberellin receptor, as it shares 63% protein sequence identity with *GID1C* from *A. thaliana*, including a conserved arginine essential for gibberellin binding activity and a mutation in the active site histidine [21]. An alignment of all BdCXEs is shown in Supplemental Figure S1.

In summary, by subtracting the gene models missing a vital part of the catalytic triad, we conclude that *B. distachyon* contains 50 gene loci potentially encoding functional carboxylesterases (Table 1).

**Table 1:** List of putative CXEs identified via protein BLAST search utilizing the MIPS PlantsDB. *BdCXE* clusters are shaded in grey. Gene models enclosed in brackets are not enclosed in the identity-based neighborhood joining tree (Supplemental Figure S5). For the RT-assay, Bd21 seedlings were incubated with 10 mg/L 3-ADON.

Gene Name	Gene Locus	*	Predicted cDNA Length (Bases)	Predicted Introns	Conserved Site Changed	Confidence Class	Results RT-Assay
*BdCXE1*	(Bradi1g06240.1)	**-**	1557	2	-	3	constitutive
	Bradi1g06241.1 ^‡^	**-**	1014	0	-	-	
*BdCXE2*	Bradi1g17780.1	**-**	1038	0	-	5	<LOD
*BdCXE3*	(Bradi1g19715.1)	**↓**	1935	3	-	3	n.t.
*BdCXE3a*	Bradi1g19713.1 ^‡^	**↓**	1032	0	-	-	n.t.
*BdCXE3b*	Bradi1g19716.1 ^‡^	**↓**	1068	0	H316del	-	n.t.
*BdCXE4*	Bradi1g19720.1	**↓**	1083	0	-	5	n.t.
*BdCXE5*	Bradi1g19730.1	**↓**	1047	0	-	4	low constitutive expression
*BdCXE6*	(Bradi1g19750.1)	**↓**	1083	0	-	5	constitutive
	Bradi1g19750.2 ^‡^	**↓**	1233	1	-	-	constitutive
*BdCXE7*	Bradi1g19760.1	**↑**	1011	0	-	4	<LOD
*BdCXE8*	Bradi1g21890.1	**-**	1038	0	-	5	n.t.
*BdCXE9*	Bradi1g38325.1 ^‡^	**-**	1053	0	-	-	-
*BdCXE10a*	(Bradi1g45925.1)	**↓**	942	0	-	5	n.t.
*BdCXE10b*	Bradi1g45921.1 ^‡^	**↓**	2643	11	-	-	-
*BdCXE11*	Bradi1g45930.1	**↑**	987	0	-	5	repressed
*BdCXE12*	Bradi1g45945.1	**-**	924	0	-	4	n.t.
*BdCXE13*	Bradi1g45960.1	**-**	999	0	-	5	induced
*BdCXE14*	Bradi1g48173.1 ^‡^	**-**	1362	3	G207S	-	-
*BdCXE15*	Bradi1g48203.1 ^‡^	**-**	1107	1	-	-	-
*BdCXE16*	Bradi1g50705.1 ^‡^	**-**	1293	1	-	-	-
*BdCXE17*	Bradi1g56807.1	**↑**	1008	0	-	4	n.t.
*BdCXE18*	(Bradi1g56817.1)	**↑**	984	0	H296R	3	n.t.
	Bradi1g56817.2 ^‡^	**↑**	990	0	-	-	n.t.
*BdCXE19*	Bradi1g56830.1	**↑**	1086	0	-	4	<LOD
*BdCXE20*	Bradi1g56860.1	**↓**	1092	0	-	4	repressed
*BdCXE21*	Bradi1g56870.1	**↑**	1017	0	-	5	low expression, repressed
*BdCXE22*	Bradi1g56910.1	**-**	1038	0	-	4	<LOD
*BdCXE23*	Bradi1g67600.1	**-**	1116	0	-	5	<LOD
*BdCXE24*	Bradi1g67930.1	**-**	999	0	S164H, H293Y	5	<LOD
*BdCXE25*	Bradi1g74240.1	**-**	984	0	D296C	5	n.t.
*BdCXE26*	Bradi2g01817.1	**-**	987	0	-	4	n.t.
*BdCXE27*	Bradi2g25470.1	**-**	1062	0	-	5	induced
*BdCXE28*	Bradi2g25600.1	**-**	1068	1	H328I	5	constitutive
*BdCXE29*	Bradi2g27300.1	**-**	1026	0	-	5	constitutive
*BdCXE30*	Bradi2g57920.1	**-**	1209	1	-	5	<LOD
*BdCXE31*	Bradi3g21747.1	**-**	1053	0	-	4	n.t.
*BdCXE32*	Bradi3g38040.1	**↑**	984	0	-	5	constitutive
*BdCXE33*	Bradi3g38045.1	**↑**	990	0	-	5	n.t.
*BdCXE34*	Bradi3g38050.1	**↑**	951	0	-	5	n.t.
*BdCXE35*	Bradi3g38060.1	**↓**	1113	0	-	5	n.t.
*BdCXE36*	Bradi3g38070.1	**↓**	1002	0	-	5	n.t.
*BdCXE37*	Bradi3g38080.1	**↑**	1119	0	-	5	<LOD
*BdCXE38*	(Bradi3g38090.1)	**↓**	1116	0	-	5	<LOD
	Bradi3g38090.2 ^‡^	**↓**	1257	0	-	-	<LOD
*BdCXE39*	Bradi3g42207.1	**-**	1089	0	H311F	4	n.t.
*BdCXE40*	Bradi3g46450.1	**↓**	951	0	-	4	constitutive
*BdCXE41*	Bradi3g46460.1	**↑**	957	0	-	4	<LOD
*BdCXE42*	Bradi4g12370.1	**-**	1032	0	-	4	n.t.
	(Bradi4g12370.1) ^‡^	**-**	957	0	D263del	-	n.t.
*BdCXE43*	Bradi4g21690.1	**-**	1065	0	-	5	<LOD
*BdCXE44*	Bradi4g21700.1	**-**	1047	0	-	5	<LOD
*BdCXE45*	Bradi4g24773.1 ^‡^	**-**	1380	0	-	-	n.t.
*BdCXE46*	Bradi4g32080.1	**-**	966	0	-	5	repressed
	Bradi4g32080.2 ^‡^	**-**	1113	0	-	-	repressed
*BdCXE47*	Bradi4g32300.1	**↓**	1221	0	-	5	repressed
*BdCXE48*	Bradi4g32310.1	**↑**	1071	0	-	5	n.t.
*BdCXE49*	Bradi4g32320.1	**↓**	954	0	-	5	low constitutive expression
*BdCXE50*	(Bradi4g32330.1)	**↓**	969	0	D264C	5	repressed
	Bradi4g32330.2 ^‡^	**↓**	1110	0	D311C		repressed
*BdCXE51*	Bradi4g32340.1	**↓**	1149	0	-	5	constitutive
*BdCXE52*	Bradi4g32350.1	**↑**	1002	0	-	5	low constitutive expression
*BdCXE53*	Bradi4g32360.1	**↓**	972	0	-	5	constitutive
*BdCXE54*	Bradi4g39410.1	**-**	1062	0	HGGdel	3	<LOD
	Bradi4g39410.2 ^‡^	**-**	1311	1	-	-	<LOD
*BdCXE55*	Bradi5g08900.1	**-**	1041	0	-	4	n.t.
*BdCXE56*	Bradi5g11800.1	**-**	942	0	-	5	n.t.

* Orientation of clustered genes: ↓, forward; ↑, reverse; LOD, limit of detection; n.t., not tested; ^‡^ Phytozome prediction, confidence classes in “MIPS PlantsDB” according to [30] (0 to 5; 5: highest probability based on available expressed sequence tags and homologous loci in other monocots).

### 2.3. Screening for BdCXEs Acting on Trichothecenes

Assuming that either constitutively-expressed or toxin-inducible genes are candidates coding for active enzymes, we started to test the expression of a subset of 33 genes by semiquantitative real-time polymerase chain reaction (RT-PCR). Designed primers for these genes are shown in Table S1. Since gene expression in cell culture is most likely highly different from that of various differentiated plant tissues, we utilized whole seedlings as a simple, but more realistic test system. Inhibition of root growth is frequently used to assess toxicity, and we therefore selected a toxin concentration still allowing root growth, but causing already clear inhibition. Bd21 seedlings were incubated with 10 mg/L of 3-ADON or 15-ADON, and cDNA was prepared. Eighteen of the 33 tested putative *BdCXE*s were expressed at a level allowing detection via RT-PCR. Interestingly, also two genes having an incomplete catalytic triad were found to be expressed (Table 1). Since most genes do not contain introns, it cannot be excluded that contaminating genomic DNA served as the PCR template. However, such contamination is unlikely, as primer pairs of genes containing an intron only yielded the smaller band expected for the cDNA, compared to the PCR product obtained with genomic DNA (see Supplementary Figure S2: *BdCXE1*, Bradi1g06240.1). Glyceraldehyde-3-phosphate dehydrogenase (GAPDH) served as positive control for expression, which was not affected by toxin treatment. In general, we observed mostly high level constitutive expression of CXE genes (see Supplementary Figure S2; *BdCXE29*; see Table 1). Some genes were rather weakly expressed (e.g., *BdCXE49*; see Supplementary Figure S2). Interestingly, some genes were induced by toxin treatment (e.g., *BdCXE13*), while others were apparently repressed (e.g., *BdCXE46*). Since a lack of expression did not allow excluding many genes, randomly, eleven candidate genes (*BdCXE1*, *13*, 27, *29*, *32*, *46*, *49*, *51*, *52*, *53* and *BdCXE54*) were chosen for functional testing by heterologous expression. As 3-ADON and 15-ADON showed instability in an unbuffered slightly alkaline *Escherichia coli* medium, yeast was chosen as the host for further characterization of the CXE candidates. In contrast to *E. coli*, yeast acidifies the synthetic complete medium, and in cultures of untransformed cells and the medium without yeast, 3-ADON and 15-ADON was stable for three days. The chosen yeast strain YZGA2274 contains deletions (*pdr5 pdr10 pdr15*) of three ATP-binding-cassette (ABC) transporter genes, which code for efflux pumps, leading to high level DON resistance of wild-type yeast strains. It contains also an inactivated trichothecene-3-*O*-acetyltransferase gene (*ayt1*). The deletion of one copy of *RPS11* (*rps11a*) further increases DON sensitivity [31]. Since grass genes are generally very GC-rich in the 5′ region of cDNAs [32] and this is often detrimental for expression in yeast [33], we expressed custom synthesized recoded genes under control of the strong and constitutive *ADH1* promoter (for recoded DNA sequences, see Supplementary Figure S2).

**Figure 2:**
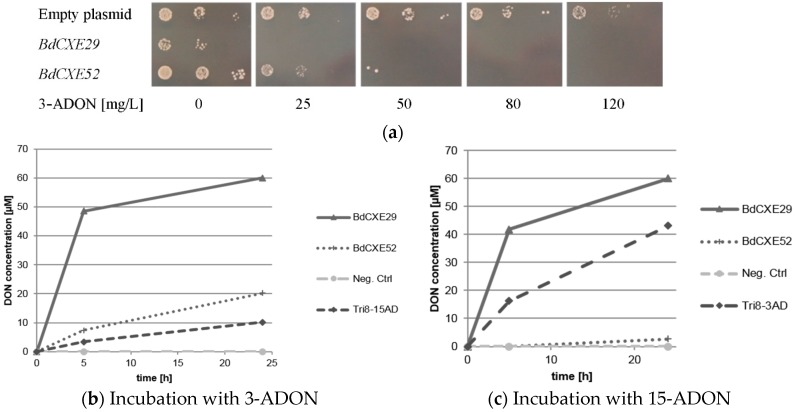
Activity of *BdCXE*s expressed in yeast strain YZGA2274 (*ayt1 pdr5*,*10*,*15 rps11a*) (**a**) expressing the indicated genes spotted in three dilutions on synthetic complete medium (SC) plates lacking uracil and containing the indicated amounts of toxin; (**b**) hydrolysis of 12 mg/L 3-acetyl-deoxynivalenol (3-ADON) into deoxynivalenol (DON) and (**c**) 15-acetyl-deoxynivalneol (15-ADON, 12 mg/L) to DON and the release into the supernatant by protein extracts of transformed yeast expressing *BdCXE29*, *BdCXE52* and Tri8 from a 15-ADON chemotype *Fusarium graminearum* strain (Tri8-15AD) or a 3-ADON chemotype strain (Tri8-3AD).

The transformed DON-sensitive yeast strain containing the empty vector was spotted on synthetic complete media lacking uracil and containing different amounts of 3-ADON. For unknown reasons, the expression of BdCXE29 led to a moderate growth reduction also on plates without toxin. As expected, 3-ADON only slightly impaired the growth of the control strain with the empty vector even at 120 mg/L in the plates. In contrast, the yeast strains expressing *BdCXE29* and *BdCXE52* were inhibited at lower concentrations (Figure 2a). This is expected when 3-ADON is converted into DON, as DON is able to efficiently block protein biosynthesis of animal ribosomes *in vitro* [10]. In contrast, towards 15-ADON, no different sensitivity was observed between the strains transformed with the empty vector and with expression vectors for *BdCXE29* or *BdCXE52*. In yeast, lower molar amounts of 15-ADON than DON are needed to inhibit growth. Most likely, this difference in toxicity is due to the better membrane permeability of the more hydrophobic acetylated toxin. At the level of the ribosomal target, DON and 15-ADON are seemingly equally inhibitory for plant and animal ribosomes *in vitro* (Supplementary Figure S3), which is most likely also true for yeast ribosomes. In contrast, 3-ADON did not elicit any translational inhibition, even at a concentration of 20 µM on animal ribosomes, but surprisingly, partly inhibited wheat ribosomes. Yet, 3-ADON was partly hydrolyzed to DON by contaminating enzymes in the wheat germ extract, as previously described [9]. In contrast to mammalian carboxylesterases, which are localized in the endoplasmic reticulum [34], BdCXE29 and BdCXE52 are both predicted to be cytosolic enzymes, so that no difference of toxicity to yeast expressing these genes is expected if the deacetylation occurs in the cytosol, and 15-ADON and its deacetylation product DON equally inhibit translation.

Protein extracts of yeast expressing *BdCXE29* and *BdCXE52* were tested if they were able to hydrolyze 3-ADON and 15-ADON to DON *in vitro* (Figure 2b,c). As a negative control, the same yeast strain transformed with the empty plasmid and for the positive control extracts of transformants expressing Tri8 proteins from either the 15-ADON or 3-ADON chemotype *F. graminearum* strains were used (see Section 3.4). Protein extracts of yeast expressing *BdCXE29* and *BdCXE52* showed deacetylating activity, while the preparations from tested strains expressing other candidate genes (*BdCXE1*, *13*, 27, *32*, *46*, *49*, *51*, *53* and *BdCXE54*) were inactive.

Furthermore, we tested the ability of yeast expressing *BdCXE29* and *BdCXE52* to convert selected acetylated types A and B trichothecenes into their corresponding deacetylated metabolite (Table 2 and Table 3). BdCXE29 rapidly deacetylated not only 12 mg/L 3-ADON and 15-ADON to DON, but also the type A trichothecenes T-2 toxin (T-2) into HT-2 toxin (HT-2) and NX-2 to NX-3. Surprisingly, BdCXE29 was only able to deacetylate the C4 residue of T-2, leading to the formation of HT-2. Further hydrolysis to T2-triol or T2-tetraol was not detected. Presumably, the isovaleryl group at the C8 prevents hydrolysis at the C15 residue of HT-2, while deacetylation of 15-ADON readily occurs. Within two hours, 100% of 10 mg/L T-2 toxin and 90% of 10 mg/L NX-2 was metabolized by a protein extract (42 µg/mL total protein) of a yeast strain expressing *BdCXE29*. Interestingly, only traces of fusarenon X (FUS-X) were deacetylated into NIV. Furthermore, the isocrotonyl residue of trichothecin (TTC) was not hydrolyzed. The best substrate for BdCXE52 was 3-ADON, followed by NX-2, 15-ADON and FUS-X (traces), but in general, conversion rates were significantly lower than for BdCXE29.

**Table 2:** Hydrolysis of acetylated trichothecenes in a yeast protein extract expressing *BdCXE29* or *BdCXE52* into the deacetylated metabolite.

Substrate	Product	BdCXE29		BdCXE52	
		2 h	24 h	2 h	24 h
3-acetyldeoxynivalenol	deoxynivalenol	77%	100%	5%	34%
15-acetyldeoxynivalenol	deoxynivalenol	70%	100%	0%	4%
T-2 toxin	HT-2 toxin	100%	100%	0%	0%
trichothecin	trichothecolone	0%	0%	0%	0%
HT-2 toxin	T-2 triol	0%	0%	0%	0%
fusarenon X	nivalenol	<1%	4%	0%	<1%
NX-2	NX-3	91%	100%	3%	17%

**Table 3:** Structure of selected trichothecenes.

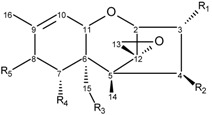	R_1_	R_2_	R_3_	R_4_	R_5_
3-acetyldeoxynivalenol	–OAc	–H	–OH	–OH	=O
15-acetyldeoxynivalenol	–OH	–H	–OAc	–OH	=O
deoxynivalenol	–OH	–H	–OH	–OH	=O
trichothecin	–H	–OIsocrot	–CH_3_	–H	=O
trichothecolone	–H	–OH	–CH_3_	–H	=O
T-2 toxin	–OH	–OAc	–OAc	–H	–OIsoval
HT-2 toxin	–OH	–OH	–OAc	–H	–OIsoval
T-2 triol	–OH	–OH	–OH	–H	–OIsoval
fusarenon X	–OH	–OAc	–OH	–OH	=O
nivalenol	–OH	–OH	–OH	–OH	=O
NX-2	–OAc	–H	–OH	–OH	–H
NX-3	–OAc	–H	–OH	–OH	–H

OIsoval, isovaleryl group; OIsocrot, isocrotonyl group.

### 2.4. Characterization of Purified BdCXE29

**Figure 3:**
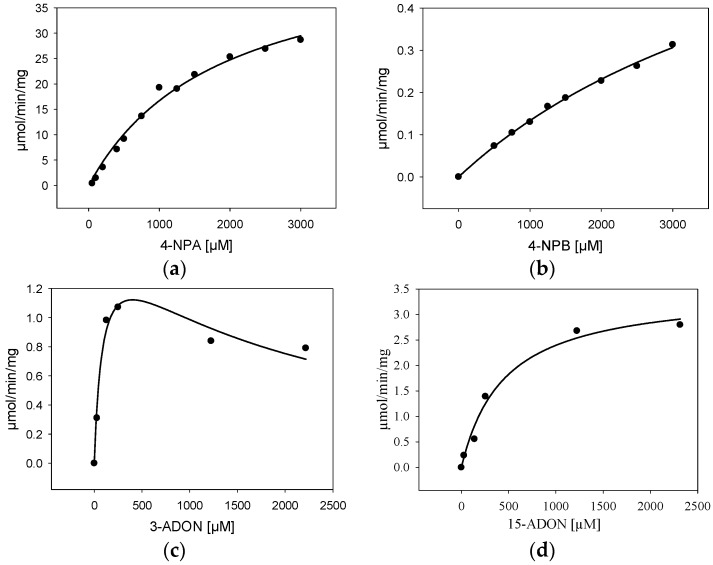
Kinetic characterization (specific activity) of BdCXE29 with (**a**) 4-nitrophenylacetate (4-NPA); (**b**) 4-nitrophenylbutyrate (4-NPB); (**c**) 3-acetyl-deoxynivalenol (3-ADON) and (**d**) 15-acetyl-deoxynivalenol (15-ADON) as the substrate.

For further characterization, *BdCXE29* was expressed in *E. coli* with an added C-terminal His_6_-tag (pET21a) and purified via one-step immobilized metal affinity chromatography (IMAC). As the protein sequence used was optimized for *S. cerevisiae*, which includes rare codons of *E. coli*, strain Rosetta 2 (DE3) containing a plasmid providing rare tRNA genes was used for expression. The IMAC fractions with the highest protein content were desalted using a PD-10 column and set to 25 mM Tris pH 7.5 yielding about 3.15 mg/mL. The one-step purified enzyme showed one intense band on the sodium dodecyl sulfate polyacrylamide gel electrophoresis (SDS-PAGE) and had a size matching the calculated weight of 38.5 kDa (Supplemental Figure S4). Kinetic assays were performed not only with the esterase standard substrates 4-nitrophenylacetate (4-NPA) and 4-nitrophenylbutyrate (4-NPB), but also with 3-ADON and 15-ADON (Table 4, Figure 3). The results for the standard substrates 4-NPA and 4-NPB clearly show that BdCXE29 favors acetates over butyrates as substrates, although BdCXE29 apparently also hydrolyzes longer side chains. The calculated Michaelis–Menten constants (*K*_m_) for 4-NPA (1860 µM) and 4-NPB (5530 µM) are significantly higher than previously-reported values for the human CES1 and CES2 [35], which are about 200 µM. The enzyme had higher affinity for 15-ADON compared to the esterase standard substrates based on the apparent *K*_m_ value of 421 µM. Assays with 3-ADON revealed substrate inhibition with increasing substrate concentration (Figure 3c). In order to obtain reliable kinetic data, regression analysis based on the kinetic model of Haldane was performed and yielded a *K*_m_ of about 89 µM 3-ADON, a *V*_max_ of 1.6 µmol/min/mg and an estimated inhibitory constant (*K_i_*) of 1800 µM, a concentration that is most likely unphysiologically high, even for cells in immediate contact with the fungus. Interestingly, the affinity to 3-ADON is considerable higher than of a recently-characterized DON-inactivating UDP-glucosyltransferase from rice [36]. Additionally, the specific activity for T-2 was determined, which was 421 ± 99 nmol/min/mg at a substrate concentration of 24 µM.

**Table 4:** Kinetic constants of BdCXE29 determined at 25 °C and pH 7.5.

Substrate	Kinetic Constant			
	*K*_m_ (µM)	*V*_max_ (µmol/min/mg)	*R*^2^	*K_i_* (µM)
4-nitrophenylacetate	1860 ± 288	48 ± 4	0.988	-
4-nitrophenylbutyrate	5530 ± 757	1.7 ± 0.2	0.996	-
15-acetyl-deoxynivalenol	421 ± 70	3.4 ± 0.2	0.973	-
3-acetyl-deoxynivalenol	89 ± 26	1.6 ± 0.2	0.939	1800 ± 490

*K_m_*, Michaelis constant; *V*_max_, maximum reaction velocity; *R*^2^, coefficient of determination; *K_i_*, inhibitory constant.

### 2.5. CXE Family of Crop Species and A. thaliana

With sequences of *BdCXE29* and *BdCXE52* at hand, the genomes of bread wheat, barley, *A. thaliana* and *B. distachyon* were mined for orthologous genes (BLASTP *E*-value threshold: 10E-10). In total, 50 putative barley CXEs and all previously-published *AtCXEs* were identified. The presence of highly similar clustered genes may indicate rapid gene amplification and gene death. Surprisingly, only 66 putative CXEs were found in hexaploid bread wheat. The comparably low number of full-length CXE genes identified in bread wheat could be the result of a loss of genes with a redundant function. By aligning all sequences utilizing the Clustal W algorithm and setting up an identity-based neighborhood joining tree, it can be seen that in most of the clades, bread wheat, barley and *Brachypodium* genes are present, including the subgroups of both active *BdCXEs*, reflecting the close relatedness between the crop species and *B. distachyon* (Supplementary Figure S5). Within the subgroup of *BdCXE29*, there are three putative barley CXEs with 65% to 68% sequence identity (AK366836, MLOC_8155.1, MLOC_8261.1) and four putative bread wheat CXEs with 59% to 70% identity (Traes_3B_CEEF8E71A.1, Traes_3B_B9EBDD0241.1, Traes_3B_84AA7E6CC.1, Traes_3B_F7CA92A7D), but all of them are either truncated (Traes_3B_B9EBDD0241.1, Traes_3B_84AA7E6CC.1) or seemingly incompletely sequenced (Traes_3B_CEEF8E71A.1, Traes_3B_F7CA92A7D) and lack the *N*-terminal sequence, including the oxyanion hole. In contrast, all three of the barley genes encode the complete catalytic triad and the oxyanion hole. The best match concerning complete sequenced bread wheat genes is Traes_6DL_8898A7FB0 with 48% identity, which also is encoding the conserved catalytic triad and the oxyanion hole, but is a member of a neighboring clade. Two bread wheat and two barley genes are part of the subgroup of *BdCXE52*. The three fully-sequenced members, AK357963, AK368846 and Traes_5BL_400181E02.1, show a sequence identity of 83% to 84%, respectively. Both, AK357963 and AK368846, which encode the exact same protein, are annotated as “gibberellin receptor GID1L2“ in the PGSB barley genome database (http://pgsb.helmholtz-muenchen.de/plant/barley/) [37], although they lack a conserved arginine essential for maintaining the gibberellin binding activity [21]. Within both clades, no *AtCXE* members can be found, and the closest homologues to *BdCXE29* and *BdCXE52* are At5g06570.1 and At3g48700.1 with 38% and 36% identity, respectively. The 20 *AtCXE* genes are distributed in seven clades and are in all, but one case, together with *B. distachyon*, barley and/or bread wheat genes. One clade consists entirely of seven *AtCXE* genes, including the 2,4-D methyl ester hydrolyzing *AtCXE12*, suggesting either that there was no common ancestral gene shared in all four species or it was lost in the lineage containing the gramineous species examined. Such a gene loss may explain the different sensitivity between broad-leafed weeds and cereals towards certain herbicides, but may also play a role in the interaction with metabolites produced by plant pathogens. In case of bread wheat, 23 of the 66 identified CXEs either have at least one mutation in the proposed catalytic triad and the oxyanion hole or are truncated genes, and four gene models are *N*-terminal incomplete, resulting in 39 to 43 putatively active bread wheat carboxylesterases. In barley, three of the 50 putative CXEs have a mutated acidic residue in the active site; one gene has no active site serine; and in three cases, there is no active site histidine in the proposed active site, resulting in 43 putative active CXEs. Interesting is also the formation of a “GID-clade”, where the CXE-related gibberellin receptors cluster. In addition to the established *GID* genes from *A. thaliana* and barley [21,38], the uncharacterized Bradi2g25600.1 (*BdCXE28*) emerges, which presumably encodes a gibberellin receptor; specifically, it shows 86% identity with the gibberellin receptor from barley, as all five genes share a conserved arginine essential for the gibberellin binding activity [21] and the fact that the active site histidine is replaced by a valine or isoleucine. Remarkable is also one clade consisting of twelve bread wheat, barley and *B. distachyon* genes all sharing an active site with a cysteine instead of the usual aspartate, suggesting that these proteins have other functions, e.g., acting as binding proteins, in a similar manner as gibberellin-binding *GID* genes, which do not all contain the active site histidine.

So far, the role of plant carboxylesterases and the consequences of the loss of gene function are unclear. On the one hand, they could function as phase I detoxification enzymes, but as discussed, also activate certain toxic compounds. For instance, deacetylated fusaproliferin (siccanol) showed higher phytotoxicity [39] than the parental compound, which is produced by many *Fusarium* species [40]. In the case of 3-ADON, deacetylation increases toxicity, while in the *Arabidopsis* system, the deacetylation of 15-ADON to DON reduced toxicity about five- to six-fold [41]. Carboxylesterases therefore may have a relevant role in the interaction of host plants with *Fusarium* and deserve more attention in resistance breeding.

## 3. Experimental Section

### 3.1. Chemicals and Reagents

Methanol (HPLC grade) was purchased from VWR (Vienna, Austria), and reverse osmosis water was further purified using an ELGA Purelab Ultra-AN-MK2 system (Veolia Water, Vienna, Austria). Formic and acetic acid, as well as ammonium acetate (both MS grade) were obtained from Sigma-Aldrich (Vienna, Austria). Ammonium formate solution (5 M) was provided by Agilent Technologies (Waldbronn, Germany), and the standard esterase substrates 4-NPA and 4-NPB where obtained from Sigma-Aldrich (Vienna, Austria). 3-ADON, 15-ADON and DON for analytical purposes were purchased from Romer Labs Diagnostic GmbH (Tulln, Austria), whereas all toxins for the treatment of cells were prepared as described previously [42]. Trichothecin was isolated from potato dextrose broth cultures of the *Trichothecium roseum* strain MA 3581 (obtained from the Austrian Center of Biological Resources, Vienna, Austria) by preparative HPLC, as described [43].

### 3.2. B. distachyon Suspension Culture Assay

For the toxin metabolism assay, *B. distachyon* Bd21 cell suspension cultures [44] were cultivated at 22 °C in the dark, re-suspended in about 83% of the original volume of fresh suspension culture medium (210 ± 50.7 mg wet weight per mL) containing Linsmaier and Skoog medium (LS) and a basal salt mix, including vitamins (Duchefa, The Netherlands), 3% (*w*/*v*) maltose, 7.5 mg/L 2,4-dichlorphenoxyacetic acid, 0.75 mg/L kinetin and set to pH 5.8. Afterwards, the medium was spiked with 75 mg/L 3-ADON or 15-ADON. With cut 5-mL tips, 1-mL samples were drawn, after “zero” (about 15 min of manipulation time), six, 24, 48, 72, 96 h post incubation (100 rpm, 21 °C in light) and transferred into 1.5 mL safe lock microcentrifuge tubes. Samples were centrifuged for five min at 4000× *g*. The supernatant was removed and diluted to 50% ethanol and stored till analysis at −20 °C. As controls, untreated suspension cultures and empty medium containing 3-ADON or 15-ADON were monitored. The concentrations of the toxins and metabolism products were determined using a liquid chromatographic-tandem mass spectrometric (LC-MS/MS) system, as described in the Section 3.7. The metabolism of each toxin was tested in three biological replicates, except for time point 96 h.

### 3.3. RNA Extraction, cDNA Synthesis and RT-PCR

The procedure for total RNA isolation was based on the TRIzol technical insert (formerly Invitrogen, now Life Technologies, Carlsbad, CA, USA). For this, six to seven *B. distachyon* seedlings were put into a flask containing 20 mL Murashige and Skoog medium (MS) and equilibrated for two days at 22 °C, shaking under light. After two days, the seedlings were transferred into ten mL of fresh MS medium containing ten mg/L 3-ADON. After zero, two, four or 24 h, samples were taken, shock frozen in liquid nitrogen and stored at −80 °C. Each sample was ground in liquid nitrogen, and 300 to 400 µL of this powder were used to isolate total RNA. After checking the RNA sample quality using native agarose gel electrophoresis and RNA quantification by spectroscopy at 260 nm, cDNA synthesis was performed according to the RevertAid H minus first strand cDNA synthesis kit (Fermentas, now Thermo Fisher Scientific, Waltham, MA, USA). For the semi-quantitative expression analysis, PCR primers designed for each individual BdCXE gene and the housekeeping gene GAPDH were tested with genomic DNA, and subsequently, the very same conditions were used for PCRs with cDNA (Table S1). The assay was repeated two times in slightly different settings (samples after 0, 2 and 4 or 24 h, two times with 3-ADON, one time with 15-ADON).

### 3.4. Cloning, Heterologous Expression in Yeast: Phenotypic Testing and Protein Extract Assays

The coding sequences of BdCXE genes Bradi1g06240.1, Bradi1g45960.1, Bradi2g25470.1, Bradi2g27300.1, Bradi3g38040.1, Bradi4g32080.1, Bradi4g32320.1, Bradi4g32340.1, Bradi4g32350.1, Bradi4g32360.1 and Bradi4g39410.1, as predicted by the MIPS PlantsDB [26], were optimized for expression in *Saccharomyces cerevisiae* by utilizing the codon usage table of *S. cerevisiae* and custom synthesized by Genewiz (South Plainfield, NJ, USA; Bradi1g45960.1) or GeneArt (now Life Technologies, Carlsbad, CA, USA; all other genes). The custom synthesized genes (DNA sequences given in Supplementary Table S2), including flanking 5′ XbaI and 3′ PstI (Bradi1g45960.1) or 5′ BamHI and 3′ HindIII (all other genes), were cloned into the multiple cloning site of yeast expression vector pVT-U [45]. Subsequently, all constructs were transformed into the hypersensitive *S. cerevisiae* strain YZGA2274 (relevant genotype: ∆pdr5::loxP ∆pdr10::hisG ∆pdr15::loxP ayt1::loxP rps11a::loxP). Strain YZGA2274 is derived from the strain YZGA515 [23], but is more toxin sensitive due to an additional deletion of the gene *RPS11A* (YDR025W), encoding a small subunit ribosomal protein [31]. Transformants were selected on synthetic complete medium lacking uracil. As positive controls, the previously-reported *TRI8* genes from *F. graminearum* [7,46] were cloned from the 15-ADON chemotype PH-1 and a 3-ADON strain (DSM No. 4258) into the same vector and transformed into YZGA2274. For the negative control, the empty vector was transformed into the same strain.

For phenotypic testing, agar plates with synthetic complete media lacking uracil containing different amounts of 3-ADON and normalized to 1.2% ethanol were used to test individual yeast transformants expressing either CXEs or solely harboring the empty vector. For this purpose, transformed strains were grown in synthetic complete media lacking uracil to the exponential phase (OD_600_ of 0.5) and diluted to OD_600_ of 0.05, 0.005 and 0.0005 with fresh selective medium. Cell suspensions (3 μL) were spotted onto plates and incubated at 30 °C for three days.

To assess protein extracts, dense liquid cultures of the yeast strains in selective medium were diluted in 100 mL synthetic complete medium lacking uracil to an OD_600_ of 0.18 and incubated overnight, shaking at 30 °C. On the next morning, an equivalent of 100 mL (OD_600_ = 1) was centrifuged at 3200× *g*; the pellet was resuspended in 600 µL water and centrifuged again in a tabletop centrifuge for 1 minute at 20,000× *g*. Glass beads (500 µL) and extraction buffer (300 µL, 50 mM Tris buffer pH 7.5, 10% glycerol) were added, and subsequently, it was vortexed seven times for 30 s and kept on ice in between. After a short centrifugation step, the supernatant was transferred to a fresh pre-cooled Eppendorf tube and centrifuged again for 15 min at 20,000× *g* at 4 °C. The supernatant was again transferred into a new precooled Eppendorf tube. Enzyme assays with the protein extract were performed as follows: 1000 µL of 100 mM potassium phosphate buffer pH 7.5 containing 10 or 20 mg/L toxin and 42 µg/mL total protein were incubated, shaking at 25 °C. Samples were taken after the indicated time points by diluting an aliquot with the same volume of acetonitrile and subsequently storing at −20 °C. Samples were analyzed according to the section liquid chromatography-tandem mass spectrometry conditions.

### 3.5. Cloning, Heterologous Expression and Purification from E. coli

Bradi2g27300.1 (*BdCXE29*) was cloned by PCR via the restriction sites NdeI and HindIII into pET21a (Merck Millipore, Vienna, Austria), including a *C*-terminal 6xHIS tag and transformed into *E. coli* Rosetta 2 (DE3). The primers used were BdCXE29-NdeI-ATG_pET21a (5′-TAT AAG CTT AGG GTT CAA GAA TCT AGC AAT-3′) and BdCXE29_C_woStop-HindIII-pET21a (5′-CAA CAT ATG ATG TCC TCT TAC ACT GCT C-3′). BdCXE29 was expressed in terrific broth supplemented with 100 mg/L ampicillin and 20 mg/L chloramphenicol in baffled flasks (2 × 500 mL), respectively. Induction with isopropyl-β-d-thiogalactopyranoside (IPTG) with a final concentration of 0.2 mM was done at an OD_600_ of about 0.7. The cells were further incubated for 24 h at 16 °C and shaking at 110 rpm. After harvesting the cell mass by centrifugation at 5500× *g*, the pellet was resuspended about 1:5 in binding buffer (50 mM phosphate, 500 mM NaCl, 20 mM imidazole, pH 7.4) and lysed by sonication. After centrifugation (30,000× *g*, 30 min), purification was performed on a Ni^2+^-charged HisTrap™ FF crude column (5 mL, GE Healthcare, Vienna, Austria) using a Äkta Purifier LC system (GE Healthcare, Vienna, Austria) by following the suppliers recommendations using non-denaturing conditions. The binding buffer (specified above) and elution buffer (same as the binding buffer, but with 500 mM imidazole) were prepared as recommended. The protein concentrations were determined via the Bio-Rad Protein Assay (Bio-Rad, Hercules, CA, USA) based on the Bradford dye-binding method. SDS-PAGE was performed with the Mini-PROTEAN system using gels with a 5% stacking and a 9% separating gel. As protein ladder PageRuler Prestained Protein Ladder was used (Thermo Fisher Scientific, Carlsbad, CA, USA). The fractions with the highest protein contents were combined, and the buffer was changed into 25 mM Tris, pH 7.5 via PD-10 columns (GE Healthcare, Vienna Austria) following the instructions of the supplier. Subsequently, the enzyme was stored in aliquots at −20 °C.

### 3.6. Enzyme Assays

The kinetic studies of BdCXE29 were performed in triplicates at 25 °C in 50 mM Tris pH 7.5. For testing, 3-ADON and 15-ADON substrate concentrations were ranging from 28 to 2320 µM. The IMAC-purified and PD-10-desalted BdCXE29 was diluted to 31.5 µg/mL, and the reaction was stopped after 60 s by the addition of 100 µL acetonitrile. Samples were measured according to the LC-MS/MS conditions described below. The kinetic studies with the standard esterase substrates 4-NPA and 4-NPB were performed by diluting BdCXE29 to 68 µg/mL and adding 50 µL substrate (4-NPA or 4-NPB ranging from 100 µM to 3000 µM). *p*-Nitrophenol released during the reaction was quantified at 400 nm in a plate reader (EnSpire 2300, PerkinElmer, Waltham, MA, USA) by reading after 55 and 58 s for 4-NPA and 4-NPB, respectively. A blank consisting of 50 µL buffer, 50 µL substrate and serial dilutions of 4-nitrophenol in 25 mM Tris pH 7.5 was included. The specific activity was calculated by utilizing the standard curve from 4-nitrophenol.

### 3.7. Liquid Chromatographic-Tandem Mass Spectrometric Conditions

Measurements concerning the deacetylation of 3-ADON and 15-ADON (Section 2.1) in *B. distachyon* Bd21 cell suspension cultures were performed as previously published for wheat [14] based on the LC-MS/MS method by Warth *et al.* [47]. In brief, a QTRAP 6500 system (Sciex, Foster City, CA, USA) interfaced with an Agilent 1290 series UHPLC system (Waldbronn, Germany) was utilized. Gradient elution with water/acetonitrile containing 20 mM ammonium acetate was performed at 30 °C using an Atlantis T3 column (3.0 × 150 mm; 3-μm particle size; Waters, Wexford, Ireland) within 14 min, operated at a flow rate of 600 μL/min. Detailed information can be found in the cited references [14,47].

All other measurements were performed on a 1290 HPLC system (Agilent Technologies) coupled to a QTrap 4000 (Sciex, Foster City, CA, USA) operated in electrospray ionization mode. Chromatographic separation was performed on a Gemini C18 (150 × 4.6 mm, 5 µm, Phenomenex, Aschaffenburg, Germany) at 25 °C and a flow rate of 0.8 mL/min. The eluents consisted of different mixtures of methanol and water (Eluent A: 20:80, *v*/*v*; Eluent B: 97:3, *v*/*v*) and contained both 5 mM ammonium acetate. Depending on the analytes, different chromatographic methods were used. ADONs/DON: 0 to 1.0 min (10% B), 1.0 to 3.0 min (10–>100% B), 3.0 to 5.5 min (100% B), 5.5 to 5.6 min (100–>10% B), 5.6 to 8.0 min (10% B); FUS-X/NIV: 0 to 1.0 min (0% B), 1.0 to 6.0 min (0–>40% B), 6.0 to 6.1 (40->100% B), 6.1 to 8.0 (100% B), 8.0 to 8.1 (100–>0% B), 8.1 to 10.0 (0% B); NX-2/NX-3: 0 to 1.0 min (0% B), 1.0 to 11.0 min (0–>100% B), 11.0 to 13.5 min (100% B), 13.5 to 13.6 min (100–>0% B), 13.6 to 16.0 min (0% B); type A trichothecenes: 0 to 1.0 min (0% B), 1.0 to 10.0 min (0–>100% B), 10.0 to 12 min (100% B), 12.0 to 12.1 min (100–>0% B), 12.1 to 15.0 min (0% B).

The QTrap 4000 was operated in the selected reaction monitoring mode, and the dwell time was set to 25 ms. The source settings were as follows: temperature 550 °C, ion spray voltage 4 kV (positive mode) and −4 kV (negative mode), curtain gas 30 psi (207 kPa of >99% nitrogen), Source Gas 1 and 2 both 50 psi (345 kPa of zero grade air), collision gas (nitrogen) set to high. The mass transitions used for the determination of the mycotoxins are displayed in Table 5. Operation of the instrument, as well as data evaluation were performed with Analyst Version 1.6.2 (Sciex, Foster City, CA, USA).

**Table 5:** Mass spectrometric parameters used for the determination of the mycotoxins.

Analyte	Precursor Ion (DP)	Quantifier (CE; CXP)	Qualifier (CE; CXP)
deoxynivalenol	355.1 (−40)	59.2 (−40; −8)	265.2 (−22; −13)
3-acetyl-deoxynivalenol	397.3 (−40)	59.2 (−38; −8)	307.1 (−20; −7)
15-acetyl-deoxynivalenol	397.3 (−40)	59.2 (−38; −8)	337.1 (−10; −7)
nivalenol	371.1 (−45)	59.1 (−42; −7)	281.1 (−22; −15)
fusarenon X	413.3.(−40)	59.1 (−40; −9)	262.9 (−22; −16)
HT-2 toxin	442.2 (46)	263.1 (21; 19)	-
	447.4 (101)	-	345.1 (27; 20)
T-2 toxin	484.3 (56)	215.2 (29; 18)	185.1 (31; 11)
T-2 tetraol	316.2 (31)	215.3 (13; 16)	281.4 (13; 8)
T-2 triol	400.2 (41)	215.2 (17; 12)	281.3 (13; 16)
NX-2	383.1 (−45)	59.0 (−36; −7)	323 (−14; −7)
NX-3	341.1 (−50)	59.0 (−36; −7)	281 (−14; −7)

DP, declustering potential; CE, collision energy; CXP, cell exit potential.

Since no analytical standard was available for trichothecolone, the measurement of the possible conversion of trichothecin to trichothecolone was performed on a 1290 UHPLC system connected to an iFunnel 6550 quadrupole time-of-flight instrument (both Agilent Technologies). For chromatographic separation, a Zorbax SB C18 Rapid Resolution High Definition column (150 mm × 2.1 mm, 1.8 µm, Agilent Technologies) was used. The eluents consisted of water (Eluent A) and methanol (Eluent B) and contained both 0.1% formic acid and 5 mM ammonium formate. The following gradient at a flow rate of 0.25 mL/min and 30 °C was applied: 0 to 0.5 min (10% B), 0.5 to 20.0 min (10–>100% B), 20.0 to 22.0 (100% B), 22.0 to 22.1 min (100–>10% B), 22.1 to 25.0 min (10% B). Mass spectrometric data were acquired between *m*/*z* 60 and 1300 at a scan rate of 3 spectra per second, and the following source conditions were used: gas temperature 130 °C; drying gas flow 14 L/min; nebulizer 30 psi (207 kPa, nitrogen); sheath gas temperature 300 °C and flow 10 L/min; capillary voltage 4 kV; nozzle voltage 0.5 kV. Data were acquired with Mass Hunter Data Acquisition Version B.05.01, and the evaluation was performed using Mass Hunter Qualitative and Quantitative Analysis Version B.06.00 (all Agilent Technologies). The extraction windows of the accurate masses of trichothecin (*m*/*z* 333.1697 [M + H]^+^; 355.1516 [M + Na]^+^) and of trichothecolone (*m*/*z* 265.1434 [M + H]^+^; *m*/*z* 282.1694 [M + NH_4_]^+^; *m*/*z* 287.1254 [M + Na]^+^) for trichothecolone were 15 ppm.

### 3.8. In Vitro Translation Assays

The assays were performed exactly as previously published [9] based on coupled *in vitro* transcription/translation systems (TnT^®^ T7 coupled Rabbit Reticulocyte Lysate System and TnT^®^ T7 Coupled Wheat Germ Extract System, both from Promega (Madison, WI, USA)). The efficiency of translation was determined by measuring the activity of the luciferase reporter using the Promega Steady-Glo^®^ Luciferase Assay System and the 2300 EnSpire®Multimode Plate Reader from Perkin-Elmer (Waltham, MA, USA).

## 4. Conclusions

In this study, we started to characterize the CXE gene family in the crop model plant *B. distachyon* and identified the first plant genes encoding enzymes with the ability to deacetylate trichothecenes. Two out of 12 genes tested by heterologous expression in yeast showed activity. The most active characterized protein, BdCXE29, has broad specificity and may have additional unknown functions besides deacetylation of trichothecenes. Hydrolysis of 3-ADON into DON increases toxicity, and consequently, CXE genes could play a role as susceptibility factors in crop plants, increasing the virulence of 3-ADON-producing *Fusarium* strains. Most likely, other still uncharacterized CXE genes with hydrolytic activity on acetylated trichothecenes may exist in *B. distachyon* and crop plants. Further characterization of this gene family with respect to the metabolism of *Fusarium* mycotoxins and other agrochemicals seems therefore warranted.

## Supplementary Materials

The following are available online at www.mdpi.com/2072-6651/8/1/6/s1.
