# Supplementary material for: Identification and Characterization of Carboxylesterases from Brachypodium distachyon Deacetylating Trichothecene Mycotoxins
Source: Toxins (Basel). 2015 Dec 25;8(1):6. doi: 10.3390/toxins8010006 (PMC4728528; doi:10.3390/toxins8010006)
Supplement: Supplementary file 1 [file toxins-08-00006-s001.zip › toxins-105210 Figure S5.pdf]

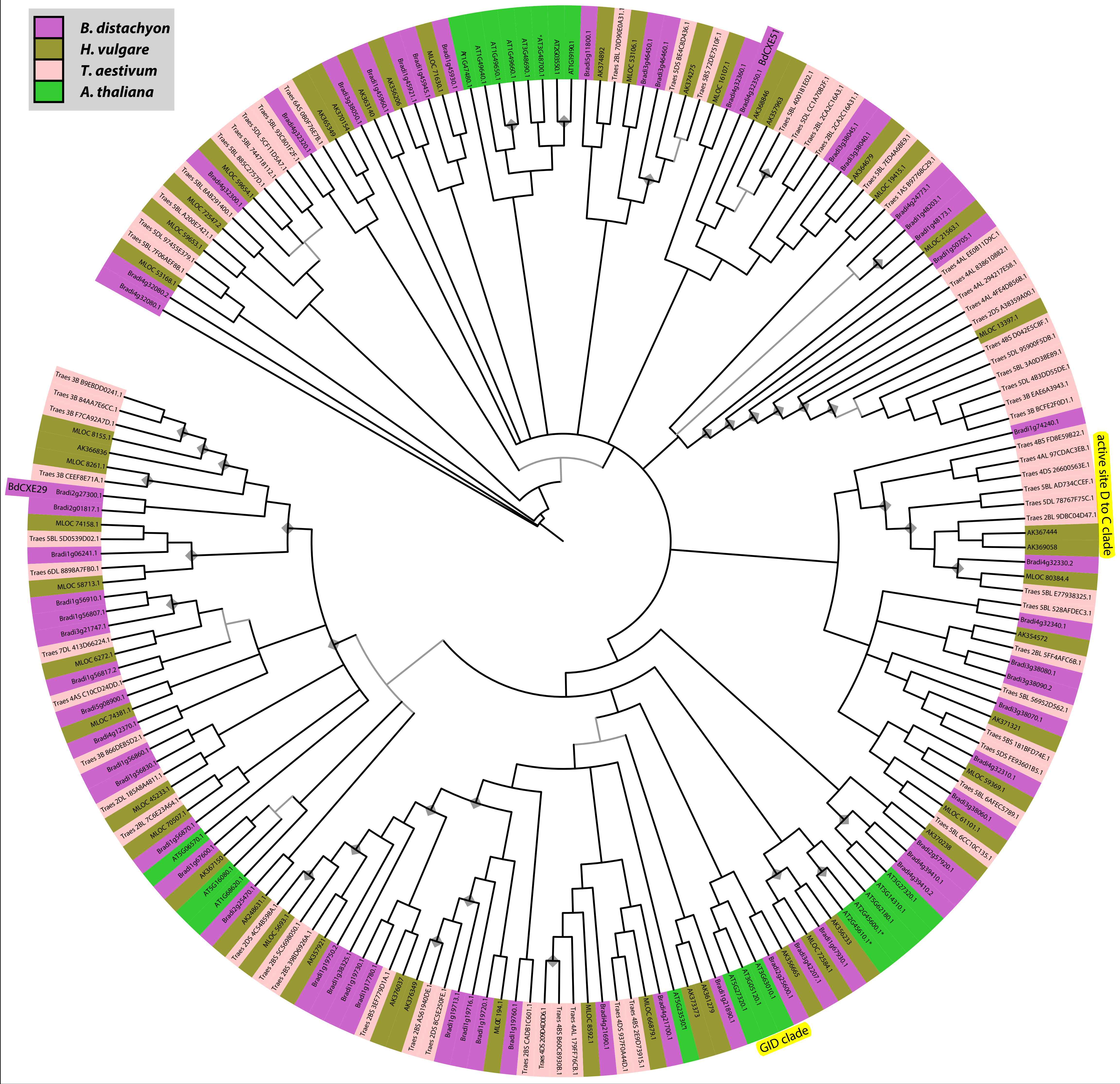

Supplemental Figure S5. Identity-based neighborhood joining tree of multi-species CXE family based on clustalW aligned sequences from a protein BLAST with BdCXE29 and BdCXE52. Bootstrap (1,000×) confidence values lower than 80 are indicated using a grey square and values lower than 60 with grey lines. AtCXEs with known activity are highlighted with a star (*).
