# Supplementary material for: Identification and Characterization of Carboxylesterases from Brachypodium distachyon Deacetylating Trichothecene Mycotoxins
Source: Toxins (Basel). 2015 Dec 25;8(1):6. doi: 10.3390/toxins8010006 (PMC4728528; doi:10.3390/toxins8010006)
Supplement: Supplementary file 1 [file toxins-08-00006-s001.zip › toxins-105210-suppl..pdf]

Supplementary Materials: Identification and Characterization of Carboxylesterases from *Brachypodium distachyon* Deacetylating Trichothecene Mycotoxins

Clemens Schmeitzl, Elisabeth Varga, Benedikt Warth, Karl G. Kugler, Alexandra Malachová, Herbert Michlmayr, Gerlinde Wiesenberger, Klaus F. X. Mayer, Hans-Werner Mewes, Rudolf Krska, Rainer Schuhmacher, Franz Berthiller and Gerhard Adam

Table S1. List of primers used for semi-quantitative expression analysis.

Gene Locus	Primer Name	Sequence (5′ to 3′)	Results RT-Assay
Bradi3g14120.3	BdGAPDH-frag-Fw	CAAGAACGACAAGACCCTCCTC	constitutive
	BdGAPDH-frag-Rv	GGACATACCGGTGAGCTTGC	
Bradi1g06240.1	B1a-fw	TAACAACAATTCAAGCCACTCACA	constitutive
	B1a-rv	TCTGCCTCGTAAGTTTTCATTGTC	
Bradi1g06240.2	B1b-fw	GAGCAACGGGACTTCACCACAG	constitutive
	B1b-rv	CTCCTTCCAGCTCGCACAAAACC	
Bradi1g17780.1	B2-fw	CGGCGGCGAGGAGAGGA	<LOD
	B2-rv	CAGCGCCAGGAAGAAGTAGAAACC	
Bradi1g19730.1	B3-fw	GCCGCCCTCCGCTTCCTCAC	low constitutive expression
	B3-rv	GTAGCGCCGGCCCCAGTCCT	
Bradi1g19750.1	B4-fw	TGGGCGCCGTCGTGGTGTCC	constitutive
	B4-rv	GTGCGCCGCCGGGTGGTTC	
Bradi1g19760.1	B5-fw	TCTCGCGGGGGTCGTCCTGAT	<LOD
	B5-rv	GCGGCCCGGTGCTCCTGG	
Bradi1g45930.1	B6-fw	GTGGCGGACATCGGCAAGGAAG	repressed
	B6-rv	TTGCGGAGGAAGCTGAGGATGC	
Bradi1g45960.1	B7-fw	CTCTCGGCGCACGGGGACAAGT	induced
	B7-rv	CACGGGCGCAGGAGATGGAACA	
Bradi1g56830.1	B8-fw	GACGCCGCCGCCCTCCTCA	<LOD
	B8-rv	GGTCCAGCGTGTCCCGGTTCATCA	
Bradi1g56860.1	B9-fw	GGCGTCGGAGGAGGCGGAGTCT	repressed
	B9-rv	CGGCGGAGGAGGCGGATGAG	
Bradi1g56870.1	B10-fw	ACCGCCCACTCAACGACCGCTACT	low expression, repressed
	B10-rv	GCTGGCCCTCGAACTCCCTGACC	
Bradi1g56910.1	B11-fw	CCCGCCGCCCACGAGGAC	<LOD
	B11-rv	CGCCGGCGGGGAACGAGA	
Bradi1g67600.1	B12-fw	GCGCCGGCGGAATCAGG	<LOD
	B12-rv	GCAGAGCGGGCCGAGACTTG	
Bradi1g67930.1	B13-fw	CGCTGGCAGGCCGGTCTTCATAAT	<LOD
	B13-rv	AGCGCGTCCACGAGCACCTTCTG	
Bradi2g25470.1	B14-fw	GCGTGGTCGGGGGCATCC	induced
	B14-rv	CGACGGCGAACTTGTTGAGGTAGA	
Bradi2g25600.1	B15-fw	CACGGCCATCTACGACCACCTCTG	constitutive
	B15-rv	GCCGCCGCTCCGACTCC	
Bradi2g27300.1	B16-fw	TTCCACGGCGGGGGCTACTGC	constitutive
	B16-rv	CGGCGGCCTGGACGGTGAC	
Bradi2g57920.1	B17-fw	TCCTGCCGCCCCGACGAG	<LOD
	B17-rv	GGCGATCCACTTAAGCACCCTGAC	
Bradi3g38040.1	B18-fw	ACCAAGATCCGCGGCCTCGTCAT	constitutive
	B18-rv	CTCGCCCTCAGCCGGTCGTAGTAG	
Bradi3g38080.1	B19-fw	CATCGCGTTGCTGGACCCCTACTT	<LOD
	B19-rv	TCCATCTCCATCGCCGCCTTCTCT	

Table S1. *Cont.*

Gene Locus	Primer Name	Sequence (5′ to 3′)	Results RT-Assay
Bradi3g38090.1	B20-fw	CGCGCTGCTCGACCCTTACTTCTG	<LOD
	B20-rv	TCCATCTCCCTCGCCGCCTTCTCT	
Bradi3g46450.1	B21-fw	GTCGCCCAACTACCACCGCTTCCT	constitutive
	B21-rv	CGCCGTGCAAACCATGACCCTCTC	
Bradi3g46460.1	B22-fw	CTCCCGGCGGCCTACGACGACT	<LOD
	B22-rv	CTCCCTCCGCCACGCACACCAT	
Bradi4g21690.1	B23-fw	CCGCTTCCTCGACAACCCCAAAAA	<LOD
	B23-rv	CCCCCGGCGGCAGGAACG	
Bradi4g21700.1	B24-fw	CGCGCCCCCGAGCACAAGTT	<LOD
	B24-rv	GTCGTAGCCGCCCACCACCAG	
Bradi4g32080.1	B25-fw	GCCGGCAGCCTACGACGACTCCT	repressed
	B25-rv	CTGGCGCCCGGGCAAATGAC	
Bradi4g32300.1	B26-fw	CCACGGCGGCGCTTTCCTCAT	repressed
	B26-rv	CTCCCCCTCGATCGCCGTGCTC	
Bradi4g32320.1	B27-fw	GCCGGCCGCATCTTCTTG	low constitutive expression
	B27-rv	GCCACGCGCTTGCCTTCAC	
Bradi4g32330.1	B28-fw	TCCGCCGACGCCGCCTACC	repressed
	B28-rv	TCGCCGTCCCACCCGCACTTC	
Bradi4g32340.1	B29-fw	TCGCGACCGGGGGAACCTCTC	constitutive
	B29-rv	CCTCTCGCCGGCCGTCTCGTA	
Bradi4g32350.1	B30-fw	GGGAGCGGCGACGGAACAAAGAAG	low constitutive expression
	B30-rv	CTCCTCCGCCTCCTCGCTGGTG	
Bradi4g32360.1	B31-fw	GCCAACATCGCGCACCACACC	constitutive
	B31-rv	TCCATCCGCCTCCCAGACCTCCAC	
Bradi4g39410.1	B32-fw	CCACTCCAACCCCGCATTCTCCAC	<LOD
	B32-rv	GGGGTACCGTTTTTGCCAGTTGC	
	B32-rv neu	ACCGCTTTTCTGGCCACATAGTCA	
Bradi5g11800.1	B33-fw	AGCGCCGGGGGAAACATCTG	<LOD
	B33-rv	CGCGCCACCGGAGGAAGTC	

<LOD below the limit of detection.

Table S2. Sequences of codon optimized genes used for heterologous expression.

Gene Name	Codon Optimized Sequence (5′ to 3′)
BdCXE1	ATGGCTTCTTCTTCTCCACCACATGTTGTTGAAGATTTGCCACCATTCTTGCAATTATTGTCCGAT<br>GGTACTGTTATCAGAGATAGATCTGCCGAATACTCTATTTTGCCAACTCCACCACCACCAGGTAG<br>ACAACCAGATGTTAGATGGAAAGATGTTGTTTACGATGCTGCTAGAGGTTTGAAGTTGAGAGTT<br>TACAAACCTCCTTTGTCCCCATCTTCATCTGGTAACAACAAAAAGTTGCCAGTCTTGGTTTACTTT<br>CACGGTGGTGGTTATGTTATCTGCTCTTTTGATTTGCCAAACTTCCACTCTTGTTGCTTGAGATTG<br>GCTGGTGAATTGCCAGCTTTGGTTTTTTCTGCTGATTATAGATTGGCCCCAGAACATAGATTACC<br>AGCTGCTTTTCATGATGCTGCTTCAGTTTTGTCTTGGGTTAGAGCACAAGCTACTGCTACTGGTAC<br>TGAAAATGCTGATCCATGGTTGGCTGATTCAGCTGATTTTTCTAGAGTTTTCGTTTCCGGTGATTC<br>TGCCGGTGGTGGTATAGTTAATCAAGTTGCTTTAAGATTGGGTTCCGGTCAATTGGATTTGGGTC<br>CATTGAGAGTTGCTGGTCATGTTATGTTGTTTCCATTATTCGGTGGTGAACAAAGAACTGCTTCTG<br>AAGCTGAATATCCACCAGGTCCACATTTGTCTTTGCCAGTTTTGGATAAGGGTTGGAGATTAGCT<br>TTACCAGTTGGTGCTACTAGAGATCATCCATTGGCTAATCCATTGGGTCCAGGTTCTCCAGCTTT<br>AGAATTGGTTGCTGGTGCTTTACCACCATTATTGGTTGTTGTTGGTGGTTTGGATTTGTTGAGAGA<br>TAGAGCTGTTGATTACGCTGCTAGATTGGAAGCTATGGGTCATGCTGTTGAATTGGTAGAATTTG<br>AAGGTCAACATCATGGTTTCTTCGCTGTTGAACCAGGTTTAGATTCTCATCCTCCAGGTGGTTTTT<br>TGTCCTTGATCAACAACAATTCCTCCCATTCTCAAGCTGTTTCTAATGGTACTTCTCCACAACCTA<br>TTAACATTGCCAATGATACCAATGGTGGTGACTCTGCTAGAACAGGTAAAAGATTGCAATGGAC<br>CAAAGAAGAAGATTGCAGATTGATTTCTGCCTGGTTGAACAATTCCAACGATCCAATTCAATCC<br>AACTACAAGAAGAACGACCAATACTGGAAGGATGTCGTTGCTGTTTATTCTTCTACTACCCCAA<br>AAGATAGAGCCAGATTGGTTAAGCAAGTTAAGGATAGATTCGGTAGAATCAAGAAAAGAGTTG<br>CTTGGTTTTGCGCCTCTTGGAAAGAAGCTAACGCCTTGTACGCTTCTGGTGAATCTGATGTTGATT<br>TGAGAGAAAGAACCATGAAGACCTATGAAGCCGATCACAAAGAAGATGGTCCATTCATCTATG<br>AACATTGCTGGGAATTCTTGAAAAAAGAACCTAAGTGGGATGCCTATTTGGAAAGATTAGAAG<br>ATTTGGAACCTAGAAGAGAATCCTCCGTCTTTATGATGAAGTGGGGTTCCATTTCCATCTGA

Table S2. *Cont.*

Gene Name	Codon Optimized Sequence (5′ to 3′)
BdCXE13	ATGGCTTCTTCTGATGCTGGTGCTGCTGGTGCTTGTGAAGCTGTTAAAATTGAATTGTTGCCTTTC<br>ATTAGGGTTTACAGGTCTGGTAGGGTCGAGAGATTGTTGGGTACTGCAACAGTCCCAGCTTCTTT<br>GGATGCTGCAACAGGTGTCGCTAGCAGGGACGTCGCTATTGACCCAGCTACTGGTGTCTCTGTC<br>AGGTTGTACTTGCCACCGGCGGCGACTTCTTCAGGTAGGGGAGGTAACAACAAGTTGCCAGTCT<br>TAGTTTATTTCCATGGAGGTGGTTTCATGGTCGAGTCTGCTGCATCTCCAACTTACCATAGATATT<br>TAAATGCTTTGGCTTCAAGAGCAGGTGTCTTGGCTGTCTCAGTTGAGTACAGAAGGGCTCCTGAA<br>CACCCATTGCCGGCGGCGTATGACGACTCTTGGGCTGCTTTGGCTTGGGCTGTCGCTGGTTGTGA<br>ATCTTACTCATCTAATCCATGGTTGTCTGCTCATGGTGACAAGTCAAGGGTCTTCTTGGCAGGTG<br>ACTCTGCTGGTGCTAACATTGCACATAACGTCGCTGCAAGAGTCGCAGCTCAAGGTTTGCCTACT<br>CCAGGTGCTGCTGCTATAGTCGGAGTCTTGTTGGTCCACCCTTACTTCTGGGACGCTTCTAACGCT<br>ATGGGTCCAGAATTGGAAACTAGAATTAGAGGTGAATGGAGATTCACATGGGCTAGGCCAGAA<br>GCTCAAGTTGACGACCCAAGGTTGTGTCCAACTTGCGCTCCAGGTGCTGCACCAAGATTGGCTG<br>CATTGCCATGCGAAAGGGTTATGGTCGCAGTTGCAGGTGAGGACTTCTTGGCTGCTAAGGGTAG<br>GGCTTACTACGCTGCTTTGTTGGCTTCAGGTTGGAGAGGTGAGGCTGAATTGGTCGATACTCCAG<br>GTCAAGGTCACGTCTTCCACTTGTTGAGACCTTGGACTGAGGCTGCTGCTGAAATGTTGGACAGA<br>GTTGCTGCTTTCATTGATAGGGCTTAA
BdCXE27	ATGGCTCCAGCTACTGAAACTCAACAACATAGAGCTACTTCTACTGGTAGAAGAAAGGTTGTTG<br>ATGAAGTTTCTGGTTGGTTGAGAGTTATGGATGATGGTACTATTGATAGAACTTGGACTGGTCCA<br>CCTGAAGCCTTGCCATTGATGCAACCAGTTGAACCATATGCTGAACCTAGAGATGGTCATACCT<br>TGCATGATTTGCCAGGTGAACCTAAATTGAGAGTTTACATTCCAGAAGCTACTGCTACAGCTAAT<br>GTTGGTTTGCCAGTTATCGTTCAATTGCATGGTGGTGGTTTCTGTATTTCTCATCCATCTTGGGTCT<br>TGTACCATCACTTTTATTCTAGATTGGCTAGAGCTTTGCCAGCTGTTGTTGTTACTGCTGAATTGC<br>CATTGGCTCCAGAACATAGATTGCCAGCTCAAATTCATACTGGTGTTGATGTCTTGCACAGATTG<br>AGATCTATTGCCTTGTCATCTGATTCCTCTTGTACTCCAGCTGAATTGTTATTGAGAGAAGCTGCT<br>GATATGTCCAGAGTTTTTTTGGTTGGTGATTCCTCAGGTGGTAATTTGGTTCATCATGTTGCTGCT<br>AGAGTTGGTGAAGATGGTCCAGATCATTGGGCTCCATTAAGAGTTGTTGGTGGTATTCCAATTCA<br>TCCAGGTTTTGTTAGAGCTGCCAGATCTAAATCTGAATTAGAACCTAGACCAGACTCCGTTTTCT<br>TCACTTTGGATATGTTGGATAAGTTCTTGGCTATGGCTTTACCAGAAGGTGCTACAAAAGATCAT<br>CCTTACACTTGTCCAATGGGTGCTGATGCTCCACCATTGGAATCTGTTCCATTGCCACCAATGTT<br>AGTTGCTGTTGGTGAACATGATTTGATCAGAGATACCAACTTGGAATACTGTGATGCTTTGAGAG<br>ATGCCGGTAAAGAAGTTGAAGTTTTGTTGTCTAAGGGTATGTCCCACTCTTTCTACTTGAACAAG<br>TTTGCTGTTGAAATGGACCCAGAAACTGGTGAAAGAACCCAAGAATTGATTGACGCCATTTCTA<br>GATTCGTTGCCAGACATTGA
BdCXE29	ATGTCCTCTTACACTGCTCCACAAGCTCAAGCTCATGTTGTTGAAGATTTTTTCGGTGTCGTCCAA<br>TTGAGATCTGATGGTTCTGTTATTAGAGGTGACGAATCTGTTTTGTTCCCACCAGAACAATATCCT<br>GAAGTTCCAGGTGTTGAATGGAAGGATGTTGTTTATCATGCTGCTCATGGTTTGAAGGCTAGAGT<br>TTACAGACCATCTTCTCCAGTTGCTGCTGAAAAAGAAGAAAAGAAGTTGCCAGTTTTGGTCTACT<br>TTCATGGTGGTGGTTACTGTTTGGGTTCTTATGCTCAACCATCTTTCCATGTTTTCTGTTTGAGAGC<br>TGCTGCAGAATTGCCAGCTGTTGTTTTGTCTGTTCAATATAGATTGGCCCCAGAACATAGATTAC<br>CAGCTGCTATTCATGATGGTGAAGGTTTTTTGTCTTGGTTGAGAGCACAAGCTGAAACTAGAAAT<br>GCTGATCCATGGTTGGCTGATTCTGCTGATTTTGCTAGAACTTTCGTTTCTGGTTGTTCTGCTGGTG<br>CTAATTTGGCTCATCATGTTACTGTTCAAGCTGCTGCTTCTTCCGGTATTATTGATTCTTCACCAGT<br>TCCATTCAGAATCGCTGGTTTTGTTTTGTTGTCTGCATTCTTCTCAGGTGTTCAAAGAACTCCAGC<br>TGAAATTGATTTGTCACCAGCTGATGTTTCTTTGACTGCTGATATGGCTGATCAATTGTGGAGAA<br>TGGCTTTGCCAGCCGGTGCTACTAGAGATCATCCATTGGCTAATCCATTTGGTCCAGAAACTGAA<br>TCCTCTGGTTTTATTGCTGCTGTTGAATTACCACCTGTTTTGGTTGTTGCTCCAGGTATTGATGTTT<br>TGAGAGATAGAGTTTTGGGTTACGCTGCTGCTATGAGAGAATTGGGTAAAGATGTTGAATTGGC<br>CAGATTCGAAGGTGAACAACATGGTTTCTCTGTTTCTAGACCATTTTCTGATGCTGCTGACGAAA<br>TGATGAGATTATTGAGAAGATTCGTCTACCAACCTAGATAA

Table S2. *Cont.*

Gene Name	Codon Optimized Sequence (5′ to 3′)
BdCXE32	ATGGCTGCTGCTCCAATGGCTCCACCACCACCAGCTGCTGATGACGAAATAGTTTATGAATCTA<br>TGCCATGCATCAGAATCTACAAGAACAGAGTCGAAAGATACTTCGGTTCCGAATTCATTGCTGC<br>TTCTACTGATGCTGCTACTGGTGTTGTTTCTAGAGATAGAACTATTTCCCCAGAAGTTTCCGCCA<br>GATTATACTTGCCAAGATTGGATGCTGATGCTCCAGCTGCTAAATTGCCAGTTTTGGTTTATTATC<br>ACGGTGGTGGTTTTTGTTTGGGTTCTGCTTTTAATCCAACCTTCCATGCCTACTTCAATTCTTTTGC<br>TGCTTTGGCTAACGTTGTTGTTGTCTCTGTTGAATATAGATTGGCTCCAGAACATCCAGTTCCAGC<br>TGCATATGCTGATTCTTGGGAAGCCTTGGCTTGGGTTGTTTCTCATGCTGCTGGTTCTGCTGGTGA<br>TGAACCTTGGTTGTCTGATCATGCTGATTTCTCTAGATTATACTTAGGTGGTGAATCTGCTGGTGC<br>TAATTTGGCTCATCATATGGCTATGAGAGTTGGTGCTGAAGGTTTAGCTCATGATACCAAAATTA<br>GAGGTTTGGTCATGATCCACCCATATTTCTTGGGTTCTAACAAGGTTGATTCCGATGATTTGGAC<br>CCAGCTACTAGAGAATCTTTAGGTTCTTTGTGGTCTGTTATGTGTCCAACTACTACTGGTGAAGA<br>TGATCCATTGATTAACCCATTTGTTGAAGGTGCTCCAGATTTGGAAGCCTTAGCATGTGGTAGAG<br>TTTTAGTTTGTGTTGCCTTGGGTGATGTTTTGAGAGACAGAGGTAGAAACTACTACGATAGATTG<br>AGAGCTTCTGGTTGGAGAGGTGAAGCTGAAATTTGGCAAGTTCCAGGTAAAGGTCATACTTTCC<br>ACTTGTTGGAACCATGTTGTGATGAAGCTGTTGCTCAAGATAAGGTTATCTCCGATTTCTTGAAC<br>AGATAA
BdCXE46	ATGGATTCTGGTTCCACCGAAGTTTTGGTTGATGCTGGTTCTTTCAGATTATACAACGATGGTCA<br>CGTTGAAAGATTGGATGGTGTTGATCATGTTCCAGCTGGTTTTGATGCTGATACTGGTGTTACTTC<br>TAAGGATGTTGTTATTGATGCCGTTACAGGTGTTGCTGCTAGATTATACTTGCCAGATATTCAAG<br>CTGCTGCTGGTAGATCTGATGGTACTGCTATTACAAAATTGCCAATCGTCGTTTTCTTCCACGGT<br>GGTTACTTTATCGTTGGTTCTGCTGGTTCACCAAGATACCATAGATACGTTAATTCTTTGGCTGCT<br>AGAGCAAGAGCTATTGCTGTTTCTGTTGATTATAGATTGGCTCCAGAACATCCATTGCCAGCTGC<br>TTATGATGATTCTTGGTTGACTTTGAATTGGGCTGCTTCTGGTTCAGCTGATCCATGGTTGTCTGA<br>ACATGGTGATTTGGGTAGAGTTTTTTTGGCTGGTTTGTCTGCTGGTGGTAATATTGCTCATAACAT<br>GGCTATTGATGCAGGTTTGACTGGTTTGAGAGCACCAGCTAGAATTGAAGGTGCTATTTTGTTGC<br>ATCCATCTTTCTGTGGTGAACAAAGAATGGAAGCAGAAGCTGAAGAACATTGGGCTTCAGTTAA<br>GAAAAGATGGGCTGTTATTTGTCCAGGTGCTAGAGGTGGTTTGGATGATCCAAGAATGAATCCA<br>ACAGCTGCTGGTGCCCCATCTTTAGCTGCTTTGGCTTGTGAAAGAATGTTAGTTACTGCTGCATC<br>TGAAGATCCTAGAATGCCAAGAGATAGAGCTTATTACGAAGCCGTTGTTTCTTCTGGTTGGGGT<br>GGTTCTGTTGAATGGTTTGTTTCTGAAGGTGAAGGTCATGGTTTCTTCATTGATGAACCAGGTGG<br>TAGTGAAGCTGCCGCTTTGA
BdCXE49	ATGGACCCAGTTCCAAAGTTGAGATTCGATTCTCCATTATTGAGAGTCTACGAAGATGGTTGCGT<br>TGAAAGATTTTTTGGTACTGATACAACTCCACCAGGTTTTGATGCTGCTACTGGTGTTACTTCTAA<br>GGATGTTGTTATTGATGGTGCCACAGGTGTTTTTGCCAGATTGTATATTCCAGACATTTGCGGTTC<br>TGGTTCCCAATCTTCTAAATTGCCAATCTTGTTGTACTTCCATGGTGGTGGTTTGGTTTTGGATTCT<br>GCTGCTTCTCCAGCTTATCATAGATACTTGAACTCCGTTGTTTCTAAGGCTGGTGTTTTGGCTATG<br>TCTGTTAATTACAGATTGGCTCCAGAACATCCAGTTCCAGCTGCTTATGATGATTCTTGGATGGC<br>TTTGGGTTGGGCTGCTTCTAGAGAAGATCCATGGTTGTCTGAACATGGTGATGCTGGTAGAATTT<br>TCTTGGCTGGTGATTCTGGTGGTGCTAATATCGTTCATAACATTGCTATTATGGCCTGCACTAGA<br>GAATATGGTTTGCCACCAGGTACTGTTTTGGAAGGTGCTATTATCTTGCATCCAATGTTCGGTGG<br>TAAAGAACCAGTTGAAGGTGAAGCTACTGAAGGTAGAGAATTTGGTGAAAAGTTGTGGTTGTTG<br>ATCATTTGCCCTGAAGGTACAGAAGGTGCTGACGATCCAAGATTGAATCCAATGGCTCATGGTG<br>CTCCATCATTGCAAAAATTGGCTTGTAGAAAGTTGTTGGTTTGCTCCGCTGAAAGAGATTTTGCT<br>AGACCAAGAGCTGCTGCTTATTATCAAGCTGTTAAGGCTTCTGCTTGGAGAGGTTCTGTTGAATG<br>GTTGGAATCTAAAGGTGAAGAACACGTATTTTTCTTGAACAAGCCAGAATCCGGTGAATCTTTG<br>GCTTTGATGGATAGAGTTGTTGCTTTTTTGGGTGGTAAGTAA

Table S2. *Cont.*

Gene Name	Codon Optimized Sequence (5′ to 3′)
BdCXE51	ATGGTCAGTAAGATCAAGAGACAATTGGCCTCTTTGCCTTTGTTTGCTAAAGCTGCTTTGTTGTTG<br>TTGATCTTGTTGTTATTATTGGCCGTCATCTTGTTGCCAATTTTCTTGATTCCACATCATCACAGAG<br>CTGAATTGCCACCAGCTTCTCCAGGTAACAACAATGGTTCTACTGGTCCAGATGATGTTGTTGCTT<br>TTGATTTCTCTCCATTCTTGGTCATGTACAAGTCTGGTAGAGTTCATAGAATGGATGGTACTGATA<br>GAGTTCCAGCTGGTGTTGATGAAGCTACTGGTGTTACTTCTAAGGATGTTGTTATCGATGGTAAG<br>ACTGGTTTGGCTGCTAGATTATACTTGCCAAGAGGTGGTGGTAAAGAAGAAGATCCAGTTTCTGG<br>TGCTTTGTTACCAGTTTTGGTTTTTTATCATGGTGGTGCCTTCGTTATTGAATCTGCTTTTACTCCAA<br>AGTACCACGTCTACTTGAATTCCTTGGTTGCTAAGGCTGGTGTAGTTGCTGTTTCTGTTGAATATA<br>GATTGGCTCCAGAACATCCATTGCCAGCTGCTTATGAAGATTCTTGGAGAGCTTTGAATTGGGTT<br>GCTAAAAATGCTGATGCTGGTCCTGAACCTTGGTTGAGAGATAGAGGTAATTTGTCCAGATTATT<br>CGTTGCTGGTGATTCTGCTGGTGCTAATATTGCTCATAATATGGCTATGAGAGCTGGTAATGAAG<br>GTGGTTTAGCTGGTGGTGCTGCTATTACTGGTATTTTGTTGTTAGATCCATACTTCTGGGGTAAAA<br>AACCTGTTGGTGCTGAAACTACTGATCAAGCTAAAAGAAGACAATACGAAGCTACCTGGTCCTT<br>CATTTGTGATGGTAAATACGGTATCGATGACCCATTGATTGATCCTTTGGCTACTCCAGCTTCTGA<br>ATTGAGAAAAATGGCTTGTGCTAGAGTTGCCGTTACTGTTTCTGGTTTGGATGATTTTGAAGAAA<br>GAGGTAAGGCTTACGCTGCTGCTTTAAGAGATTCAGGTTGGGATGGTGAAGTTGTTCAATACGAA<br>ACTGCTGGTGAAAGACACGTTTACTTTTTGGATGCTCCAAAGAATCCAAAGTCCGCTAAAGAATT<br>GGCTTTTGCTGCTGGTTATTTGTCTAGAGAATGA
BdCXE52	ATGGCTGGTTCTGGTGCTTCTAATGATGAAGTTGTTTTGGAAATCGAACACTGCATCAGAATCTTC<br>AAGTCTGGTAGAGTTGAAAGATACTTCGGTTCTGATCCAATTCCACCATCTACTGATGCTTCTACT<br>GGTGTTGCTTCTAAGGATAGAACTATCTCTCCAGATGTTGCCGTCAGATTATACTTGCCACCAGTT<br>GCTGCTACTGGTTCAGGTGATGGTACTAAGAAATTGCCTTTGTTGGTTTACTTTCACGGTGGTGGT<br>TTTGTTTTACACACTGCTTTTAACGCTGTTTTCCATGCTTATTTGGCTTCATTGGCTGCTAGAGCTA<br>GAGCAATAGTTGTTTCTGTTGATTACAGATTGGCTCCAGAACATCCATTGCCAGCTGCTTACGAA<br>GATTCTTGGAGAGCTTTGGTTTGGGCTGCTTCTCATGCTTCTGGTGGTTCATGTTGTGGTACTTCAG<br>AAGAAGCTGAAGAAGAACCTTGGTTGACTGAACATGCTGATTTCTCCAGATTATTCTTGGGTGGT<br>GAATCTGCTGGTGCTAATATTGCTCATCATATGGCTATGAGAGCTGGTACTGATAGATTGCCAGC<br>CGGTGCTTCAATTTCTGGTATAGTTTTGGTTCACCCATACTTTTTGGGTCATGGTAAAGTTCCATCC<br>GAAGATTCAGATCCAGTTATGGCTGAAAACGTTGTTAAGATGTGGCATGTTGTTAGACCAACTAC<br>TACAGGTGTTGATGATCCTTGGATTAACCCATTGGCAGCTGGTGCTCCACCAATGAGAGGTTTGG<br>CTTGCGGTAGAGTTTTGATGTGTTTGGCTGAAACTGATGTCTGTAGAGATAGAGGTAGAGCTTATT<br>GTGAAGGTTTGATGGCTTCTGGTTGGGCTGGTGAAGTTGAAGTTTTGGAAGTTGCTGGTCAAGGT<br>CATTGCTTTCATTTGGGTAATTTCACCTGTGATGATGCCGTTAGACAAGATGATGCTATTGCTAGA<br>TTCTTGAACCCTTAA
BdCXE53	ATGTCTGACGCTGATGCTGGTGGTGACGAAGTCATACACGACGCTCCAAACTTCATAAGGGTCTA<br>CAAGTCTGGAAGGGTCGAGAGGTTCTTGCCAGTCGACTTCGCTCCACCTTCAATAGACCCAACTA<br>CAGGAGTCTCTTCTAAGGACGTCCCAATATTGCCTGGTGCAGGAGTCTCTGCAAGGATTTACTTG<br>CCAGCAGCTCCTGCTGGTGGTCATCAGTCAAAGGTCCCAGTCTTGTTGTTCTTCCACGGTGGAGG<br>ATTCTGCTTGGGTTCTGCATTCGACGAGGCTGTCCACGGTCACGCAAACCAATTGTCTGCACAGG<br>CTTCAGTTATTGTCGTCTCAGTCGAGTACAGATTGGCACCAGAACATCCTGTCCCAGCATTGTAC<br>GAGGACGCATGGGCTGCATTGCAATGGGTCGCTGCTCATGCGGCGGGGCAGGGACCTGAGCCTT<br>GGTTGACAGCTCACGCTGACTTTGGTAGGGTTCACGTCGGTGGTGAGTCTGCTGGTGCTAACATA<br>GCACACCACACAGCAATGAGAGCAGGTGTCGAGGAATTGGGACACGGAGTCAAAGTCAACTCA<br>TTGGTCTTGATTCACCCATACTTCTTGGGAGGAGATTCTTCAGAGTCAGACGAGATGGGTATGGC<br>TTTGTTGAGGGAGTTGGTCAGGTTGTGGCCTGTTGTCTGCCCTGGTACTTCTGGTTGCGACGACCC<br>ATGGATTAACCCAATGTCAGACGGTGCTCCTTCTTTGGCAGGTTTAGGTTGTGCAAGGGCATTGG<br>TCTGCGTCGGAGGAAAGGACGCTATGAGGGGAAGGGGTAGGTTGTACTGCGAGAAGTTGATGG<br>GTTCTGGTTGGCATGGTGAGGTTGAGGTTTGGGAGGCAGACGGTCAGGGTCACGGTTTCCACTTG<br>TTCTGCCCAACATCAACACAGACTAAGGCACAGGTCAGAGTCATAACTGACTTTATGTCTAGGTAAC

Table S2. *Cont.*

Gene Name	Codon Optimized Sequence (5′ to 3′)
BdCXE54	ATGCCATCTGTTACCGTCAAGTTGTACTCCTTGATTTTCAAGTTGTTGTTGAGAAGAAGATTGTCC<br>TCCTTGTCTGTTTCTGATCCAGCTCCAGCTGGTGCAGCTTCTTCTTTTGGTGTTTCTTCTAGACCAG<br>CTGATCATCATCCATCTCCACATTCTAATCCAGCTTTTTCTACTGCTGCTCCAGATGCTGTTGCTA<br>CAAAAGACTTGCATCCAGATCCATTGTCATCCTTGCATTTGAGATTATTCTTGCCAAACCCACAT<br>CATGCTACTCCATTGAACAATCCACCACCACCTCCATTGAGAAGATCATCTTTTCCAGAAAGAG<br>GTTGTAAGGGTAACTGGCAAAAAAGATATCCAGCTGCTTTTGAAGATGGTGTCACTGTTTTGAG<br>ATGGATTGCTAAGCAAGCTAATTTGGCTGCTTGTGGTAGAATGATGGCTAAAGGTGCTGGTACTT<br>GTGGTACTGATTCATTTGGTGCTGCTATGGTTGAACCTTGGTTGGCTGCTCATGCTGATCCATCAA<br>GATGTGTTTTGTTGGGTGTTTCATGTGGTGCTAACATTGCTGATTACGTTGCTAGAAAAGCTGTTG<br>AAGCTGGTAAGTTTTTGGACCCAGTTAAGGTTGTTGCTCAAGTTTTGATGTACCCATTCTTCATGG<br>GTTCTTCTCCAACTCAATCTGAATTGAAGTTGGCTAACTCTTACTTCTACGATAAGTCTACCTGTT<br>TGTTGGCTTGGAAGTTGTTTTTGCCTGAAGATGAATTCTGCTTGGATCATCCAGCAGCTAATCCAT<br>TATTGCCAGGTAGAGGTCCACCATTGAAATTGATGCCACCAACTTTGACTATCGTTGCTGAATTG<br>GATTGGATGAAGGATAGAGCTATTGCCTACTCTGAAGAATTGAGAAAGGTTAACGTTGATGCCC<br>CAGTTTTGGAATACAAAGATGCCGTTCATGAATTCGCTACTTTGGACGGTTTGTTGAAAACTCCT<br>GAAGCTCAAGCCTGTGCTGAAGATATTGCTATTTGGGTTAAGAAGTACATCTCCTTGAGAGGTC<br>ACGAATTCTCTTACTGA

Figure S1

Figure S2. Clustal W alignment of all putative BdCXEs. Green bars below the consensus sequence indicate conserved features in the order: HGG box, GXSXG motif, active site acidic residue and active site histidine.

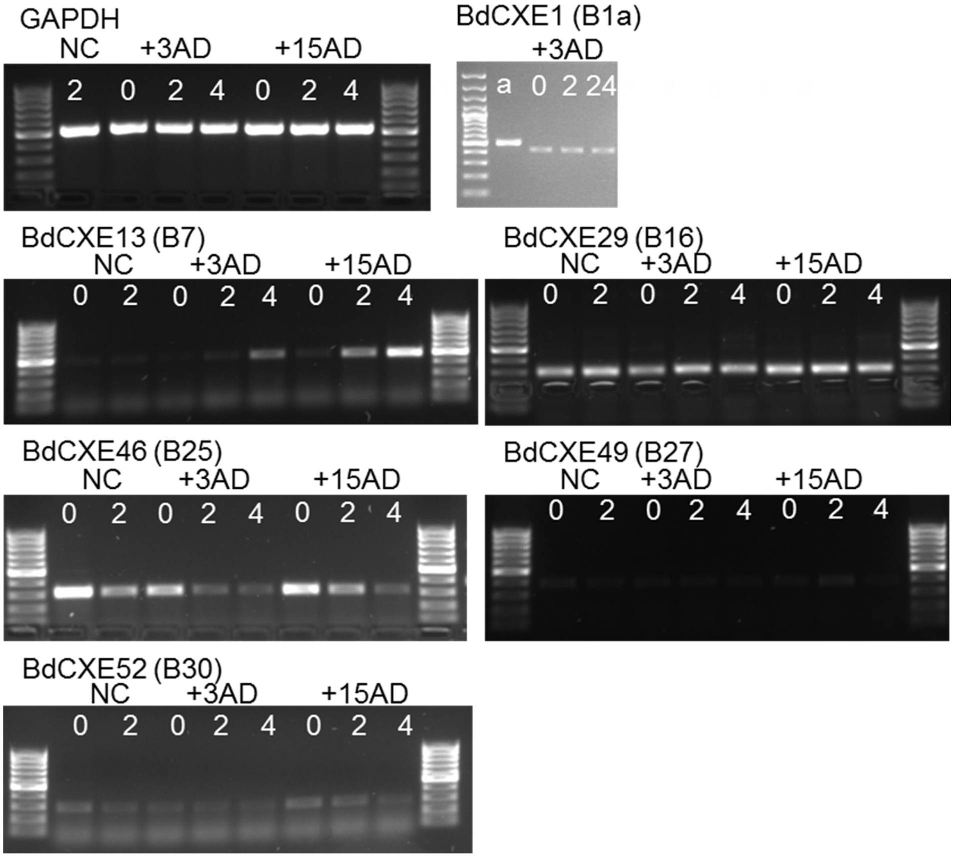

Figure S2. Real time polymerase chain reaction (RT-PCR) results for selected genes (in brackets the primer code according to Supplemental Table S2). NC indicates mock treated samples, the white numbers show the time point (h) of sampling after the indicated treatment, and (a) indicates PCR with BdCXE1a primers enclosing an intron using genomic DNA. As Marker GeneRuler 100 bp DNA ladder (LifeTechnologies, Carlsbad, CA, USA) was used.

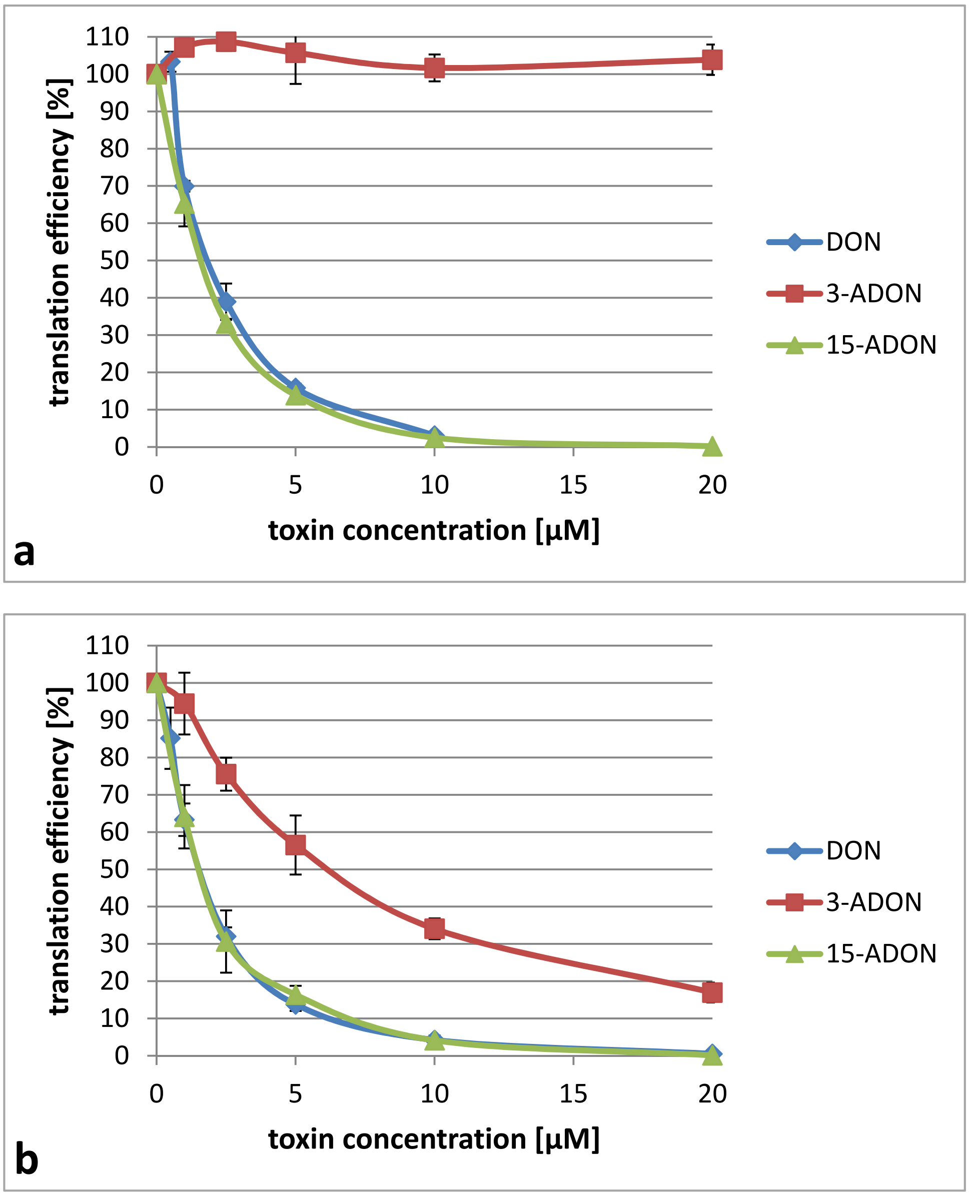

Figure S3. *In vitro* translation inhibition assays of the indicated toxins (**a**) rabbit reticulocyte lysate (**b**) wheat germ extract.

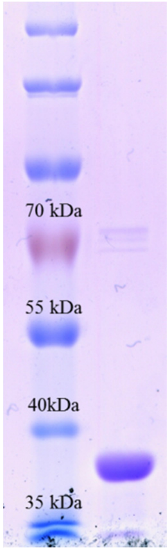

Figure S4. Sodium dodecyl sulfate polyacrylamide gel electrophoresis (SDS-PAGE) of the one step purified BdCXE29. Lane 1 PageRuler Prestained Protein ladder (Life Technologies, Carlsbad, CA, USA); Lane 2 IMAC purified BdCXE29 (BdCXE29-cHis6, 38.5 kDa).
