# Supplementary material for: Identification and Characterization of Carboxylesterases from Brachypodium distachyon Deacetylating Trichothecene Mycotoxins
Source: Toxins (Basel). 2015 Dec 25;8(1):6. doi: 10.3390/toxins8010006 (PMC4728528; doi:10.3390/toxins8010006)
Supplement: Supplementary file 1 [file toxins-08-00006-s001.zip › toxins-105210 Figure S1.pdf]

```
                     1        10        20        30        40        50        60        70        80        90        100       110       120       130
Consensus            MSCRAAPWSSPSHPRGRPFPASARLRTPFGHMHVWKNPILKNLSRIGGVPMMFGLPSGLFRCMASSSTGNGGFSRPSSTDETPVPVYSWPDKQRPRVCILGGGFGGLYTALRLESLVWPGNNKPQVLLVD

1. Bradi1g06241.1
2. Bradi1g17780.1
3. Bradi1g19713.1
4. Bradi1g19716.1
5. Bradi1g19720.1
6. Bradi1g19730.1
7. Bradi1g19750.2
8. Bradi1g19760.1
9. Bradi1g21890.1
10. Bradi1g38325.1
11. Bradi1g45921.1   MSCRAAPWSSPSHPRGRPFPASARLRTPFGHMHVWKNPILKNLSRIGGVPMMFGLPSGLFRCMASSSTGNGGFSRPSSTDETPVPVYSWPDKQRPRVCILGGGFGGLYTALRLESLVWPGNNKPQVLLVD
12. Bradi1g45930.1
13. Bradi1g45945.1
14. Bradi1g45960.1
15. Bradi1g48173.1
16. Bradi1g48203.1
17. Bradi1g50705.1
18. Bradi1g56807.1
19. Bradi1g56817.2
20. Bradi1g56830.1
21. Bradi1g56860.1
22. Bradi1g56870.1
23. Bradi1g56910.1
24. Bradi1g67600.1
25. Bradi1g67930.1
26. Bradi1g74240.1
27. Bradi2g01817.1
28. Bradi2g25470.1
29. Bradi2g25600.1
30. Bradi2g27300.1
31. Bradi2g57920.1
32. Bradi3g21747.1
33. Bradi3g38040.1
34. Bradi3g38045.1
35. Bradi3g38050.1
36. Bradi3g38060.1
37. Bradi3g38070.1
38. Bradi3g38080.1
39. Bradi3g38090.2
40. Bradi4g12370.1
41. Bradi4g21690.1
42. Bradi4g21700.1
43. Bradi4g24773.1
44. Bradi4g32080.1
45. Bradi4g32080.2
46. Bradi4g32300.1
47. Bradi4g32310.1
48. Bradi4g32320.1
49. Bradi4g32330.2
50. Bradi4g32340.1
51. Bradi4g32350.1
52. Bradi4g32360.1
53. Bradi4g39410.1
54. Bradi4g39410.2
55. Bradi3g42207.1
56. Bradi3g46450.1
57. Bradi3g46460.1
58. Bradi5g08900.1
59. Bradi5g11800.1
```

```
                        140       150       160       170       180       190       200       210       220       230       240       250       260
Consensus               QSDRFVFKPMLYELLSGEVDVWEIAPYFTELLKNTSVQFVKDSVKLLRPSDHLRREPGGSCTGGVVHLESGTVIEYDWLVLALGAEAKIDVVPGSAEHAIPFTTLEHALKVESELKMLERRRFGKSSPPI

1. Bradi1g06241.1
2. Bradi1g17780.1
3. Bradi1g19713.1
4. Bradi1g19716.1
5. Bradi1g19720.1
6. Bradi1g19730.1
7. Bradi1g19750.2
8. Bradi1g19760.1
9. Bradi1g21890.1
10. Bradi1g38325.1
11. Bradi1g45921.1        QSDRFVFKPMLYELLSGEVDVWEIAPYFTELLKNTSVQFVKDSVKLLRPSDHLRREPGGSCTGGVVHLESGTVIEYDWLVLALGAEAKIDVVPGSAEHAIPFTTLEHALKVESELKMLERRRFGKSSPPI
12. Bradi1g45930.1
13. Bradi1g45945.1
14. Bradi1g45960.1
15. Bradi1g48173.1
16. Bradi1g48203.1
17. Bradi1g50705.1
18. Bradi1g56807.1
19. Bradi1g56817.2
20. Bradi1g56830.1
21. Bradi1g56860.1
22. Bradi1g56870.1
23. Bradi1g56910.1
24. Bradi1g67600.1
25. Bradi1g67930.1
26. Bradi1g74240.1
27. Bradi2g01817.1
28. Bradi2g25470.1
29. Bradi2g25600.1
30. Bradi2g27300.1
31. Bradi2g57920.1
32. Bradi3g21747.1
33. Bradi3g38040.1
34. Bradi3g38045.1
35. Bradi3g38050.1
36. Bradi3g38060.1
37. Bradi3g38070.1
38. Bradi3g38080.1
39. Bradi3g38090.2
40. Bradi4g12370.1
41. Bradi4g21690.1
42. Bradi4g21700.1
43. Bradi4g24773.1
44. Bradi4g32080.1
45. Bradi4g32080.2
46. Bradi4g32300.1
47. Bradi4g32310.1
48. Bradi4g32320.1
49. Bradi4g32330.2
50. Bradi4g32340.1
51. Bradi4g32350.1
52. Bradi4g32360.1
53. Bradi4g39410.1
54. Bradi4g39410.2
55. Bradi3g42207.1
56. Bradi3g46450.1
57. Bradi3g46460.1
58. Bradi5g08900.1
59. Bradi5g11800.1
```

	270–390
Consensus	EVAIVGLGYSGVELAATISERLKNTGTVKAINVQTTICPSAPPGNRDAALKVLESQNIQLFLGYSVSCVREFYASDDSSSMATDEKEAGGDHKKLLVELQPAQRGLQSQVLEADLVLWTVGSTSQIPRLQ
1. Bradi1g06241.1	
2. Bradi1g17780.1	
3. Bradi1g19713.1	
4. Bradi1g19716.1	
5. Bradi1g19720.1	
6. Bradi1g19730.1	
7. Bradi1g19750.2	
8. Bradi1g19760.1	
9. Bradi1g21890.1	
10. Bradi1g38325.1	
11. Bradi1g45921.1	EVAIVGLGYSGVELAATISERLKNTGTVKAINVQTTICPSAPPGNRDAALKVLESQNIQLFLGYSVSCVREFYASDDSSSMATDEKEAGGDHKKLLVELQPAQRGLQSQVLEADLVLWTVGSTSQIPRLQ
12. Bradi1g45930.1	
13. Bradi1g45945.1	
14. Bradi1g45960.1	
15. Bradi1g48173.1	
16. Bradi1g48203.1	
17. Bradi1g50705.1	
18. Bradi1g56807.1	
19. Bradi1g56817.2	
20. Bradi1g56830.1	
21. Bradi1g56860.1	
22. Bradi1g56870.1	
23. Bradi1g56910.1	
24. Bradi1g67600.1	
25. Bradi1g67930.1	
26. Bradi1g74240.1	
27. Bradi2g01817.1	
28. Bradi2g25470.1	
29. Bradi2g25600.1	
30. Bradi2g27300.1	
31. Bradi2g57920.1	
32. Bradi3g21747.1	
33. Bradi3g38040.1	
34. Bradi3g38045.1	
35. Bradi3g38050.1	
36. Bradi3g38060.1	
37. Bradi3g38070.1	
38. Bradi3g38080.1	
39. Bradi3g38090.2	
40. Bradi4g12370.1	
41. Bradi4g21690.1	
42. Bradi4g21700.1	
43. Bradi4g24773.1	
44. Bradi4g32080.1	
45. Bradi4g32080.2	
46. Bradi4g32300.1	
47. Bradi4g32310.1	
48. Bradi4g32320.1	
49. Bradi4g32330.2	
50. Bradi4g32340.1	
51. Bradi4g32350.1	
52. Bradi4g32360.1	
53. Bradi4g39410.1	
54. Bradi4g39410.2	
55. Bradi3g42207.1	
56. Bradi3g46450.1	
57. Bradi3g46460.1	
58. Bradi5g08900.1	
59. Bradi5g11800.1	

	400–520
Consensus	PPDAPYVIPLNGRGQVETEETLQVKGHPRTFAIGDSAALRDPSGKFLPANAQVAFQQADFAGWNIWAAINDRPLLPFRFQNLGEMMXJXXXXXXIXXXXXXXLXLSKSSXNXARXXXSLXXXXXXNXXAX
1. Bradi1g06241.1	
2. Bradi1g17780.1	
3. Bradi1g19713.1	
4. Bradi1g19716.1	
5. Bradi1g19720.1	
6. Bradi1g19730.1	
7. Bradi1g19750.2	MTTPFFTQASDDKLSDVSIQIALPM
8. Bradi1g19760.1	
9. Bradi1g21890.1	
10. Bradi1g38325.1	
11. Bradi1g45921.1	PPDAPYVIPLNGRGQVETEETLQVKGHPRTFAIGDSAALRDPSGKFLPANAQVAFQQADFAGWNIWAAINDRPLLPFRFQNLGEMMTLGRNDAAITASFIEGLTLEGPLGHAARKLVYCLRMPTDEHRVK
12. Bradi1g45930.1	
13. Bradi1g45945.1	
14. Bradi1g45960.1	
15. Bradi1g48173.1	
16. Bradi1g48203.1	
17. Bradi1g50705.1	
18. Bradi1g56807.1	
19. Bradi1g56817.2	
20. Bradi1g56830.1	
21. Bradi1g56860.1	
22. Bradi1g56870.1	
23. Bradi1g56910.1	
24. Bradi1g67600.1	
25. Bradi1g67930.1	
26. Bradi1g74240.1	
27. Bradi2g01817.1	
28. Bradi2g25470.1	
29. Bradi2g25600.1	
30. Bradi2g27300.1	
31. Bradi2g57920.1	
32. Bradi3g21747.1	
33. Bradi3g38040.1	
34. Bradi3g38045.1	
35. Bradi3g38050.1	
36. Bradi3g38060.1	
37. Bradi3g38070.1	
38. Bradi3g38080.1	
39. Bradi3g38090.2	MIPASPPSICRPGRLKLVLDRSNSALTRKVASVIQSLLLYSTTE
40. Bradi4g12370.1	
41. Bradi4g21690.1	
42. Bradi4g21700.1	
43. Bradi4g24773.1	
44. Bradi4g32080.1	
45. Bradi4g32080.2	MDESKLSPNYLIPSWPLVLTCPANLIHA
46. Bradi4g32300.1	MRARAQRLICSSVNTPMPFFSMAIRSGANIWAG
47. Bradi4g32310.1	
48. Bradi4g32320.1	
49. Bradi4g32330.2	MPIPVANSAR
50. Bradi4g32340.1	MVSKIKRQLASLPLFAKAALLLL---I
51. Bradi4g32350.1	
52. Bradi4g32360.1	
53. Bradi4g39410.1	
54. Bradi4g39410.2	
55. Bradi3g42207.1	
56. Bradi3g46450.1	
57. Bradi3g46460.1	
58. Bradi5g08900.1	
59. Bradi5g11800.1	

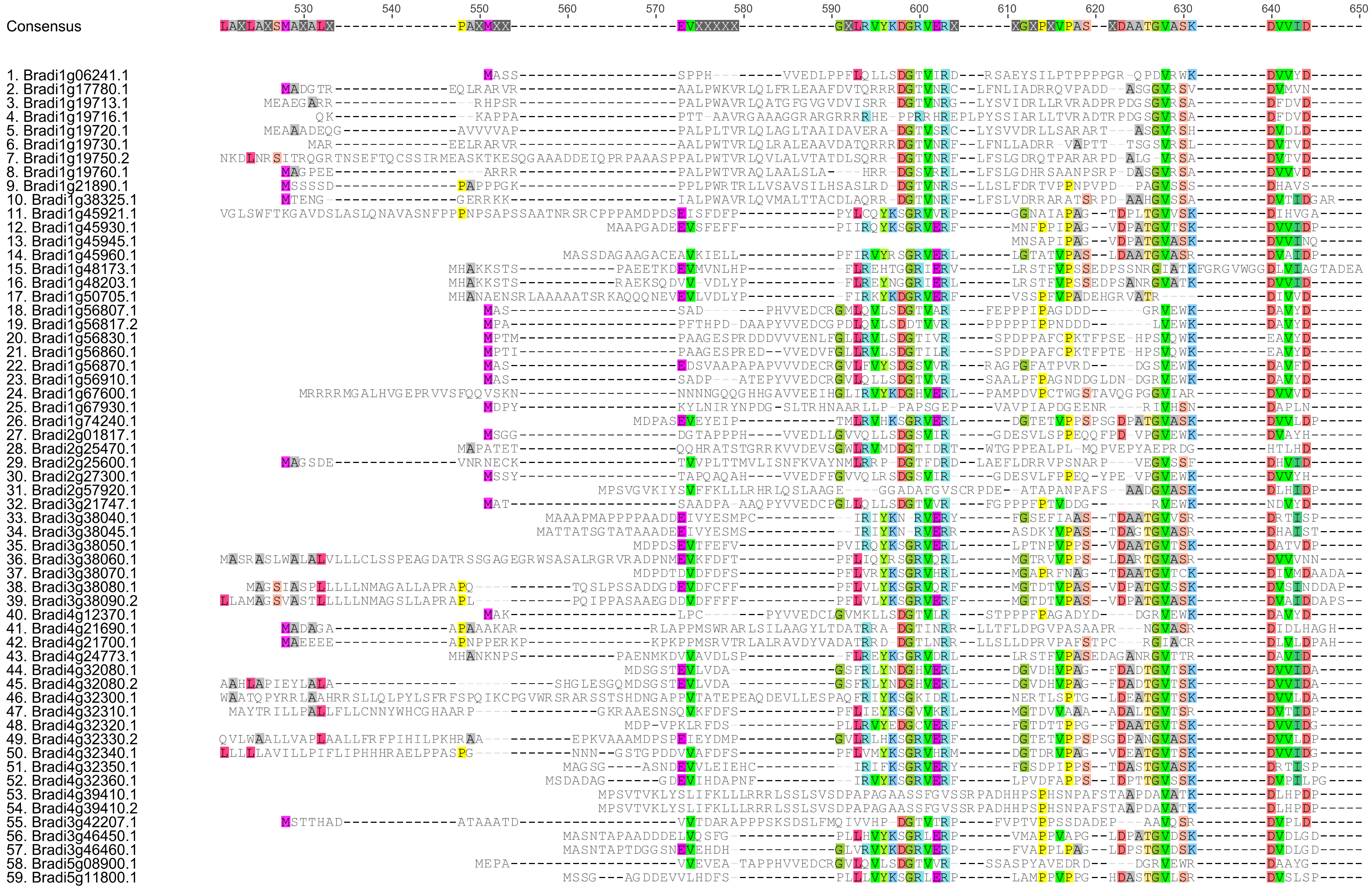
530
540
550
560
570
580
590
600
610
620
630
640
650
Consensus
1. Bradi1g06241.1
2. Bradi1g17780.1
3. Bradi1g19713.1
4. Bradi1g19716.1
5. Bradi1g19720.1
6. Bradi1g19730.1
7. Bradi1g19750.2
8. Bradi1g19760.1
9. Bradi1g21890.1
10. Bradi1g38325.1
11. Bradi1g45921.1
12. Bradi1g45930.1
13. Bradi1g45945.1
14. Bradi1g45960.1
15. Bradi1g48173.1
16. Bradi1g48203.1
17. Bradi1g50705.1
18. Bradi1g56807.1
19. Bradi1g56817.2
20. Bradi1g56830.1
21. Bradi1g56860.1
22. Bradi1g56870.1
23. Bradi1g56910.1
24. Bradi1g67600.1
25. Bradi1g67930.1
26. Bradi1g74240.1
27. Bradi2g01817.1
28. Bradi2g25470.1
29. Bradi2g25600.1
30. Bradi2g27300.1
31. Bradi2g57920.1
32. Bradi3g21747.1
33. Bradi3g38040.1
34. Bradi3g38045.1
35. Bradi3g38050.1
36. Bradi3g38060.1
37. Bradi3g38070.1
38. Bradi3g38080.1
39. Bradi3g38090.2
40. Bradi4g12370.1
41. Bradi4g21690.1
42. Bradi4g21700.1
43. Bradi4g24773.1
44. Bradi4g32080.1
45. Bradi4g32080.2
46. Bradi4g32300.1
47. Bradi4g32310.1
48. Bradi4g32320.1
49. Bradi4g32330.2
50. Bradi4g32340.1
51. Bradi4g32350.1
52. Bradi4g32360.1
53. Bradi4g39410.1
54. Bradi4g39410.2
55. Bradi3g42207.1
56. Bradi3g46450.1
57. Bradi3g46460.1
58. Bradi5g08900.1
59. Bradi5g11800.1

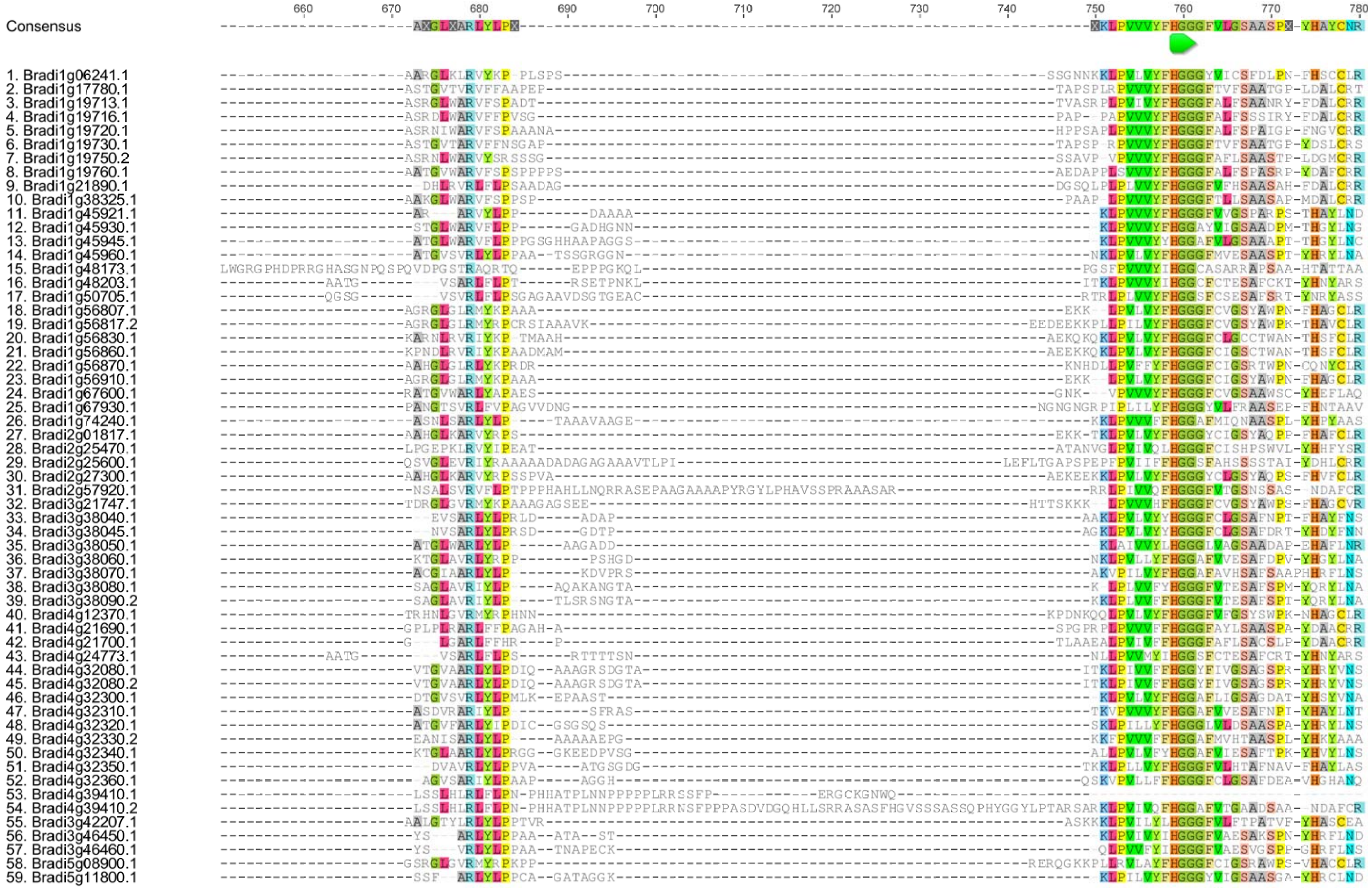
660
670
680
690
700
710
720
730
740
750
760
770
780
Consensus
1. Bradi1g06241.1
2. Bradi1g17780.1
3. Bradi1g19713.1
4. Bradi1g19716.1
5. Bradi1g19720.1
6. Bradi1g19730.1
7. Bradi1g19750.2
8. Bradi1g19760.1
9. Bradi1g21890.1
10. Bradi1g38325.1
11. Bradi1g45921.1
12. Bradi1g45930.1
13. Bradi1g45945.1
14. Bradi1g45960.1
15. Bradi1g48173.1
16. Bradi1g48203.1
17. Bradi1g50705.1
18. Bradi1g56807.1
19. Bradi1g56817.2
20. Bradi1g56830.1
21. Bradi1g56860.1
22. Bradi1g56870.1
23. Bradi1g56910.1
24. Bradi1g67600.1
25. Bradi1g67930.1
26. Bradi1g74240.1
27. Bradi2g01817.1
28. Bradi2g25470.1
29. Bradi2g25600.1
30. Bradi2g27300.1
31. Bradi2g57920.1
32. Bradi3g21747.1
33. Bradi3g38040.1
34. Bradi3g38045.1
35. Bradi3g38050.1
36. Bradi3g38060.1
37. Bradi3g38070.1
38. Bradi3g38080.1
39. Bradi3g38090.2
40. Bradi4g12370.1
41. Bradi4g21690.1
42. Bradi4g21700.1
43. Bradi4g24773.1
44. Bradi4g32080.1
45. Bradi4g32080.2
46. Bradi4g32300.1
47. Bradi4g32310.1
48. Bradi4g32320.1
49. Bradi4g32330.2
50. Bradi4g32340.1
51. Bradi4g32350.1
52. Bradi4g32360.1
53. Bradi4g39410.1
54. Bradi4g39410.2
55. Bradi3g42207.1
56. Bradi3g46450.1
57. Bradi3g46460.1
58. Bradi5g08900.1
59. Bradi5g11800.1

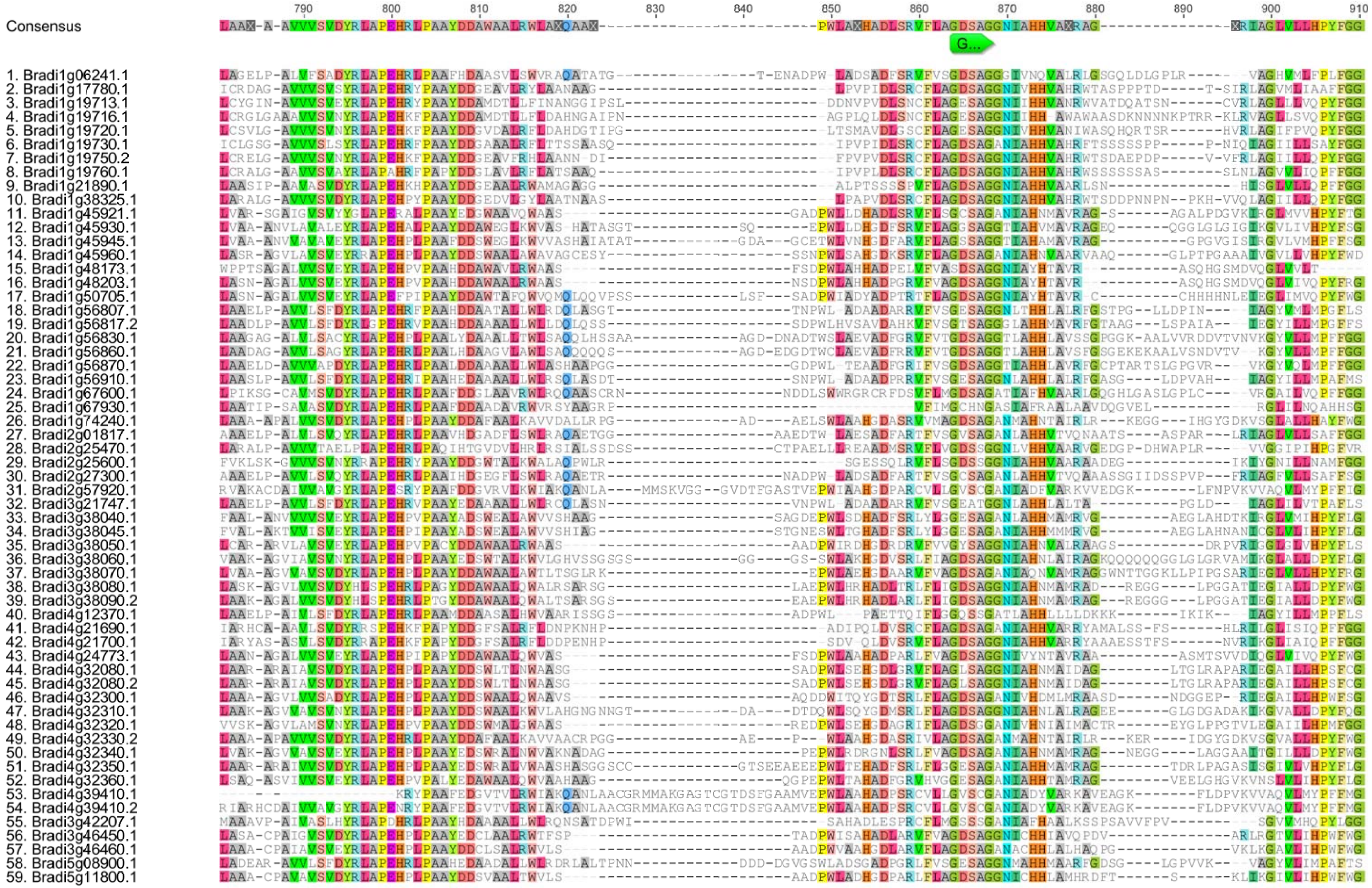
790
800
810
820
830
840
850
860
870
880
890
900
910
Consensus
G...
1. Bradi1g06241.1
2. Bradi1g17780.1
3. Bradi1g19713.1
4. Bradi1g19716.1
5. Bradi1g19720.1
6. Bradi1g19730.1
7. Bradi1g19750.2
8. Bradi1g19760.1
9. Bradi1g21890.1
10. Bradi1g38325.1
11. Bradi1g45921.1
12. Bradi1g45930.1
13. Bradi1g45945.1
14. Bradi1g45960.1
15. Bradi1g48173.1
16. Bradi1g48203.1
17. Bradi1g50705.1
18. Bradi1g56807.1
19. Bradi1g56817.2
20. Bradi1g56830.1
21. Bradi1g56860.1
22. Bradi1g56870.1
23. Bradi1g56910.1
24. Bradi1g67600.1
25. Bradi1g67930.1
26. Bradi1g74240.1
27. Bradi2g01817.1
28. Bradi2g25470.1
29. Bradi2g25600.1
30. Bradi2g27300.1
31. Bradi2g57920.1
32. Bradi3g21747.1
33. Bradi3g38040.1
34. Bradi3g38045.1
35. Bradi3g38050.1
36. Bradi3g38060.1
37. Bradi3g38070.1
38. Bradi3g38080.1
39. Bradi3g38090.2
40. Bradi4g12370.1
41. Bradi4g21690.1
42. Bradi4g21700.1
43. Bradi4g24773.1
44. Bradi4g32080.1
45. Bradi4g32080.2
46. Bradi4g32300.1
47. Bradi4g32310.1
48. Bradi4g32320.1
49. Bradi4g32330.2
50. Bradi4g32340.1
51. Bradi4g32350.1
52. Bradi4g32360.1
53. Bradi4g39410.1
54. Bradi4g39410.2
55. Bradi3g42207.1
56. Bradi3g46450.1
57. Bradi3g46460.1
58. Bradi5g08900.1
59. Bradi5g11800.1

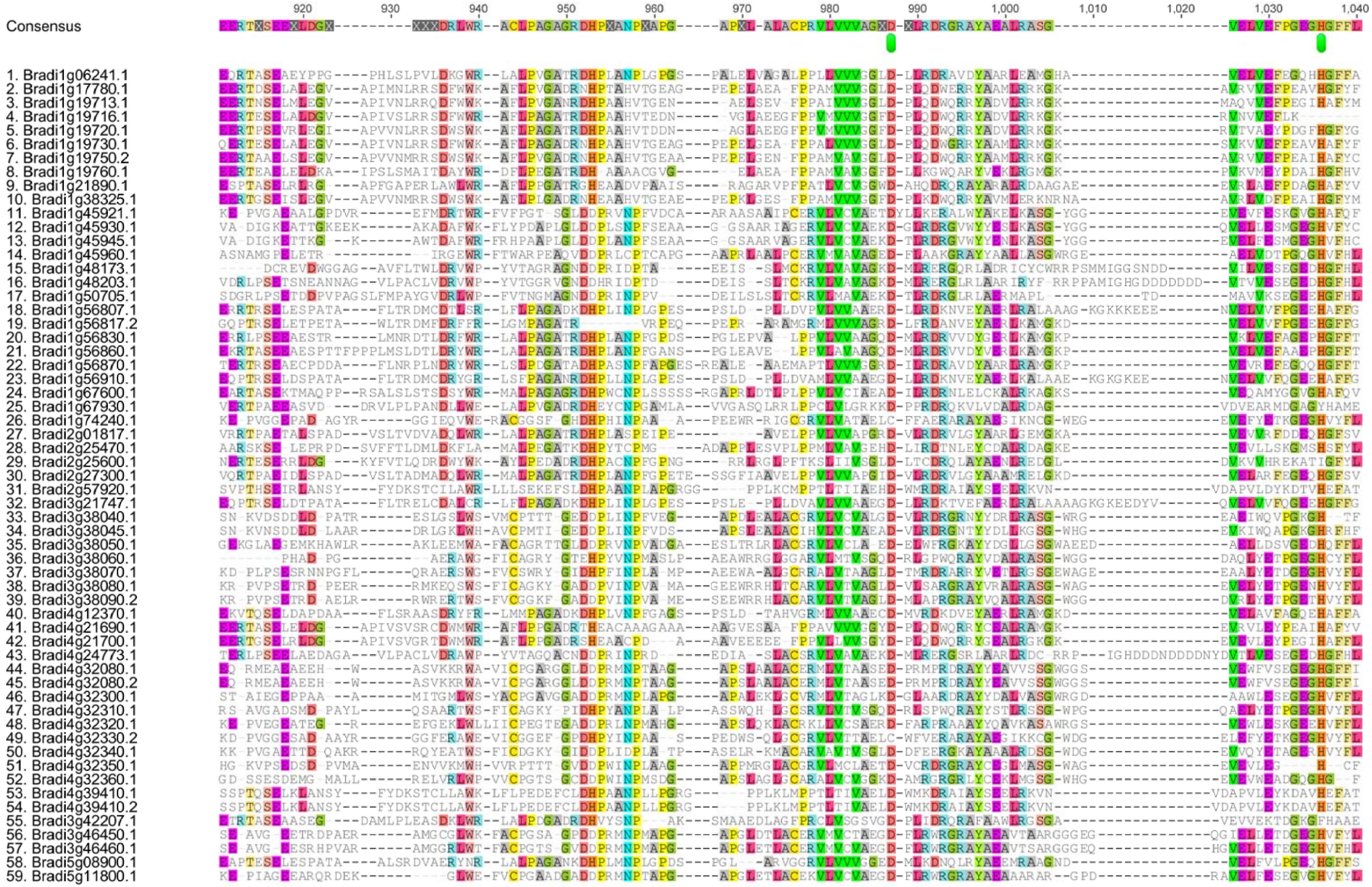
920
930
940
950
960
970
980
990
1,000
1,010
1,020
1,030
1,040
Consensus
1. Bradi1g06241.1
2. Bradi1g17780.1
3. Bradi1g19713.1
4. Bradi1g19716.1
5. Bradi1g19720.1
6. Bradi1g19730.1
7. Bradi1g19750.2
8. Bradi1g19760.1
9. Bradi1g21890.1
10. Bradi1g38325.1
11. Bradi1g45921.1
12. Bradi1g45930.1
13. Bradi1g45945.1
14. Bradi1g45960.1
15. Bradi1g48173.1
16. Bradi1g48203.1
17. Bradi1g50705.1
18. Bradi1g56807.1
19. Bradi1g56817.2
20. Bradi1g56830.1
21. Bradi1g56860.1
22. Bradi1g56870.1
23. Bradi1g56910.1
24. Bradi1g67600.1
25. Bradi1g67930.1
26. Bradi1g74240.1
27. Bradi2g01817.1
28. Bradi2g25470.1
29. Bradi2g25600.1
30. Bradi2g27300.1
31. Bradi2g57920.1
32. Bradi3g21747.1
33. Bradi3g38040.1
34. Bradi3g38045.1
35. Bradi3g38050.1
36. Bradi3g38060.1
37. Bradi3g38070.1
38. Bradi3g38080.1
39. Bradi3g38090.2
40. Bradi4g12370.1
41. Bradi4g21690.1
42. Bradi4g21700.1
43. Bradi4g24773.1
44. Bradi4g32080.1
45. Bradi4g32080.2
46. Bradi4g32300.1
47. Bradi4g32310.1
48. Bradi4g32320.1
49. Bradi4g32330.2
50. Bradi4g32340.1
51. Bradi4g32350.1
52. Bradi4g32360.1
53. Bradi4g39410.1
54. Bradi4g39410.2
55. Bradi3g42207.1
56. Bradi3g46450.1
57. Bradi3g46460.1
58. Bradi5g08900.1
59. Bradi5g11800.1

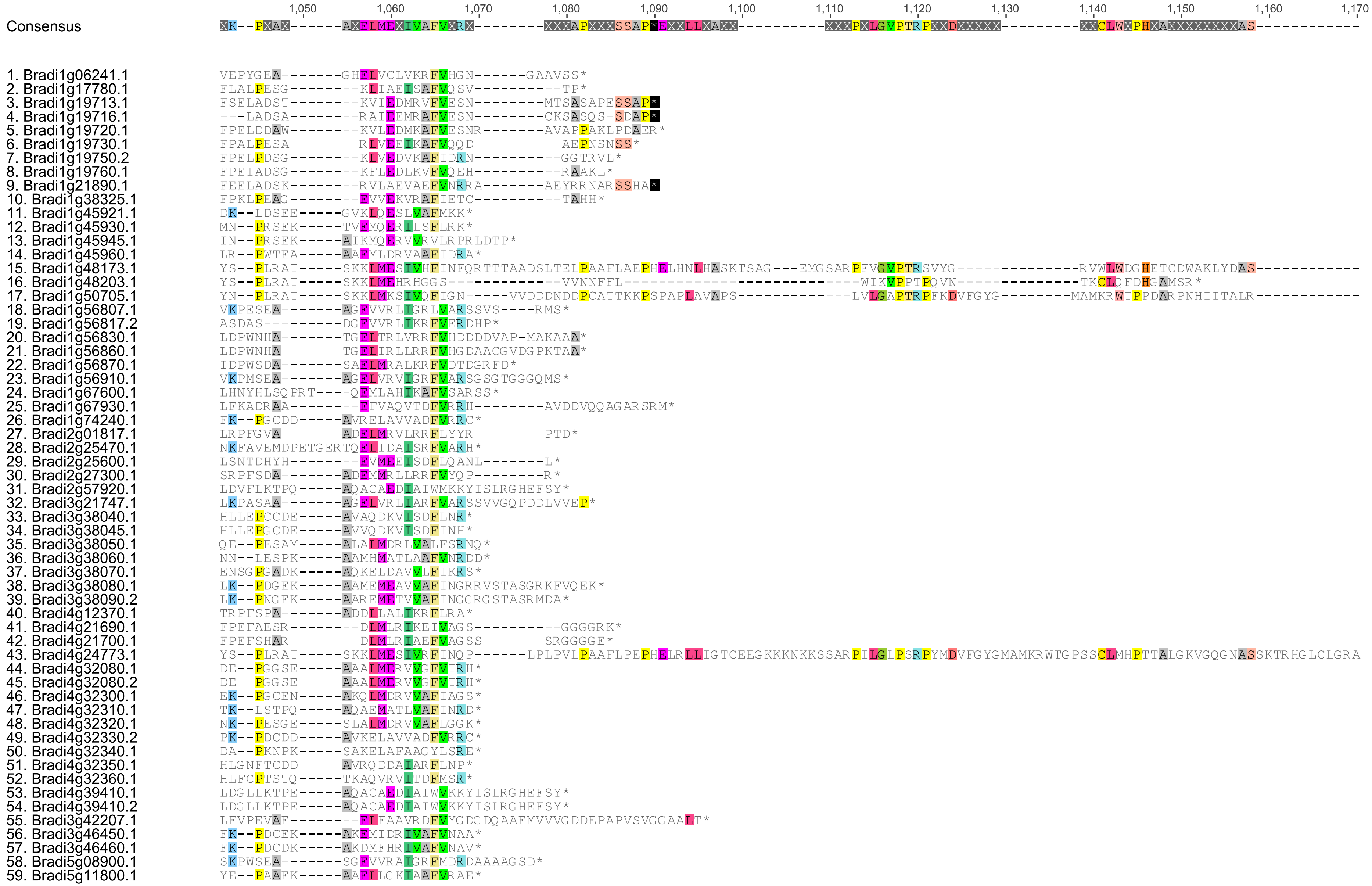
1,050
1,060
1,070
1,080
1,090
1,100
1,110
1,120
1,130
1,140
1,150
1,160
1,170
Consensus
1. Bradi1g06241.1
2. Bradi1g17780.1
3. Bradi1g19713.1
4. Bradi1g19716.1
5. Bradi1g19720.1
6. Bradi1g19730.1
7. Bradi1g19750.2
8. Bradi1g19760.1
9. Bradi1g21890.1
10. Bradi1g38325.1
11. Bradi1g45921.1
12. Bradi1g45930.1
13. Bradi1g45945.1
14. Bradi1g45960.1
15. Bradi1g48173.1
16. Bradi1g48203.1
17. Bradi1g50705.1
18. Bradi1g56807.1
19. Bradi1g56817.2
20. Bradi1g56830.1
21. Bradi1g56860.1
22. Bradi1g56870.1
23. Bradi1g56910.1
24. Bradi1g67600.1
25. Bradi1g67930.1
26. Bradi1g74240.1
27. Bradi2g01817.1
28. Bradi2g25470.1
29. Bradi2g25600.1
30. Bradi2g27300.1
31. Bradi2g57920.1
32. Bradi3g21747.1
33. Bradi3g38040.1
34. Bradi3g38045.1
35. Bradi3g38050.1
36. Bradi3g38060.1
37. Bradi3g38070.1
38. Bradi3g38080.1
39. Bradi3g38090.2
40. Bradi4g12370.1
41. Bradi4g21690.1
42. Bradi4g21700.1
43. Bradi4g24773.1
44. Bradi4g32080.1
45. Bradi4g32080.2
46. Bradi4g32300.1
47. Bradi4g32310.1
48. Bradi4g32320.1
49. Bradi4g32330.2
50. Bradi4g32340.1
51. Bradi4g32350.1
52. Bradi4g32360.1
53. Bradi4g39410.1
54. Bradi4g39410.2
55. Bradi3g42207.1
56. Bradi3g46450.1
57. Bradi3g46460.1
58. Bradi5g08900.1
59. Bradi5g11800.1

	1,180 1,190 1,195
Consensus	-------XXFTXXXTGSXXXXXXX*
1. Bradi1g06241.1	
2. Bradi1g17780.1	
3. Bradi1g19713.1	
4. Bradi1g19716.1	
5. Bradi1g19720.1	
6. Bradi1g19730.1	
7. Bradi1g19750.2	
8. Bradi1g19760.1	
9. Bradi1g21890.1	
10. Bradi1g38325.1	
11. Bradi1g45921.1	
12. Bradi1g45930.1	
13. Bradi1g45945.1	
14. Bradi1g45960.1	
15. Bradi1g48173.1	-------WCFTESWTGECIQDKAL*
16. Bradi1g48203.1	
17. Bradi1g50705.1	--------VATPHETGSPYP*
18. Bradi1g56807.1	
19. Bradi1g56817.2	
20. Bradi1g56830.1	
21. Bradi1g56860.1	
22. Bradi1g56870.1	
23. Bradi1g56910.1	
24. Bradi1g67600.1	
25. Bradi1g67930.1	
26. Bradi1g74240.1	
27. Bradi2g01817.1	
28. Bradi2g25470.1	
29. Bradi2g25600.1	
30. Bradi2g27300.1	
31. Bradi2g57920.1	
32. Bradi3g21747.1	
33. Bradi3g38040.1	
34. Bradi3g38045.1	
35. Bradi3g38050.1	
36. Bradi3g38060.1	
37. Bradi3g38070.1	
38. Bradi3g38080.1	
39. Bradi3g38090.2	
40. Bradi4g12370.1	
41. Bradi4g21690.1	
42. Bradi4g21700.1	
43. Bradi4g24773.1	RPGQTYMGSFSAAGHGSSVIKNFF*
44. Bradi4g32080.1	
45. Bradi4g32080.2	
46. Bradi4g32300.1	
47. Bradi4g32310.1	
48. Bradi4g32320.1	
49. Bradi4g32330.2	
50. Bradi4g32340.1	
51. Bradi4g32350.1	
52. Bradi4g32360.1	
53. Bradi4g39410.1	
54. Bradi4g39410.2	
55. Bradi3g42207.1	
56. Bradi3g46450.1	
57. Bradi3g46460.1	
58. Bradi5g08900.1	
59. Bradi5g11800.1	
